# An In Silico Analysis of PCR-Based Monkeypox Virus Detection Assays: A Case Study for Ongoing Clinical Surveillance

**DOI:** 10.3390/v15122327

**Published:** 2023-11-27

**Authors:** Kuncheng Song, Hayden N. Brochu, Qimin Zhang, Jonathan D. Williams, Lakshmanan K. Iyer

**Affiliations:** 1 Center of Excellence for Bioinformatics, Data Science and AI, Laboratory Corporation of America Holdings (Labcorp), Burlington, NC 27215, USA; songk2@labcorp.com (K.S.); brochuh@labcorp.com (H.N.B.); zhangq3@labcorp.com (Q.Z.); 2 Labcorp Research and Development, Laboratory Corporation of America Holdings (Labcorp), Burlington, NC 27215, USA; willj89@labcorp.com

**Keywords:** monkeypox virus, monkeypox, poxvirus, quantitative polymerase chain reaction, in silico analysis

## Abstract

The 2022 global Mpox outbreak swiftly introduced unforeseen diversity in the monkeypox virus (MPXV) population, resulting in numerous Clade IIb sublineages. This propagation of new MPXV mutations warrants the thorough re-investigation of previously recommended or validated primers designed to target MPXV genomes. In this study, we explored 18 PCR primer sets and examined their binding specificity against 5210 MPXV genomes, representing all the established MPXV lineages. Our results indicated that only five primer sets resulted in almost all perfect matches against the targeted MPXV lineages, and the remaining primer sets all contained 1–2 mismatches against almost all the MPXV lineages. We further investigated the mismatched primer-genome pairs and discovered that some of the primers overlapped with poorly sequenced and assembled regions of the MPXV genomes, which are consistent across multiple lineages. However, we identified 173 99% genome-wide conserved regions across all 5210 MPXV genomes, representing 30 lineages/clades with at least 80% lineage-specific consensus for future primer development and primer binding evaluation. This exercise is crucial to ensure that the current detection schemes are robust and serve as a framework for primer evaluation in clinical testing development for other infectious diseases.

## 1. Introduction

Monkeypox virus (MPXV) is a DNA virus classified under the genus *Orthopoxvirus,* to which other well-known viruses are classified, such as variola virus (the virus that causes smallpox) and vaccinia virus (the virus used for variola vaccine development) [1]. While MPXV is primarily transmitted among non-human mammals (especially monkeys and rodents), there is documented evidence of human-to-human transmission [1,2]. The MPXV-associated disease has since been designated by the World Health Organization (WHO) as Mpox, formerly monkeypox [3]. Before 2022, Mpox was an endemic public health concern in central and western Africa, with rare and isolated cases reported in North America, Asia, and Europe [4,5,6]. In May 2022, Mpox cases were reported in several European countries and quickly spread to numerous regions across the globe [7]. By the end of July 2023, the 2022 Mpox outbreak had 88,600 laboratory-confirmed cases and 152 deaths [8,9]. This 2022 Mpox outbreak peaked in July 2022, and the number of reported cases decreased significantly in 2023. However, there were 360 and 495 confirmed cases globally in June and July of 2023, respectively [8], indicating that sustained monitoring of the spread and evolution of MPXV is needed.

In ongoing public health emergencies, fast and accurate diagnostic tools are critical for controlling and monitoring the situations, and polymerase chain reaction (PCR)-based methods are frequently offered as a detection assay for laboratory confirmations. DNA viruses, such as MPXV, do not mutate as freely as RNA viruses (such as SARS-CoV-2) [10], but the mutation rate of Mpox has increased from 1–2 mutations per genome per year to approximately 50 mutations per year as of 2019 [11]. Recent comparisons between the 2022 MPXV and related MPXV strains from 2018/2019 revealed a 6–12 fold increase in mutation rate [12,13]. These mutations could alter the virus’ epidemiology and pathogenicity and impact the specificity and sensitivity of PCR-based detection assays. Currently, the evolution of MPXV clades and their refined lineages is tracked by Nextstrain [14], and the classification of MPXV genomes is possible using Nextclade [15]. Two main clades of MPXV genomes exist: Clade I and Clade II. Clade I is the Congo Basin (Central Africa) clade with the RefSeq genome NC_003310.1 based on a sample collected in 1996, and Clade II is the West African clade with the RefSeq genome NC_063383.1 based on a sample collected in 2018. Clade II is further subdivided into IIa and IIb, and the IIb lineages were the cause of the 2022 Mpox outbreak. However, the MPXV samples we analyzed in this study contain 2022–2023 samples from both Clade I and Clade IIa, highlighting the need to examine all MPXV lineages. In this study, we used non-discriminatory nomenclature to address the MPXV lineages [16], i.e., Clade I and Clade II.

Multiple MPXV PCR-based assays designed to detect Clade I and II were developed and validated before the 2022 Mpox outbreak. These primers were designed using one or a few MPXV genomes and cross-examined across orthopoxviral and poxviral genomes both computationally and experimentally [17,18,19,20,21,22,23,24,25,26,27,28]. However, these primer or probe binding sites likely require re-evaluation, as MPXV polymorphisms have continued to be documented in Nextstrain [14] since these assay developments. Previous studies have confirmed the presence of mismatches and the negative impact on the binding efficiency even with a single mismatch [29,30,31,32,33], including those affecting MPXV primers [31]. To our knowledge, no lineage-specific investigations of MPXV primers have been reported.

To examine the binding efficiencies of known primer sets designed to target MPXV, we evaluated 5210 MPXV genomes gathered from the NCBI viral genomes resource [34] (access date: 21 May 2023) as well as the lineage information from both the Nextstrain hMPXV (NC_063383.1) and MPXV (NC_003310.1) databases. We evaluated 18 PCR primer sets designed to target MPXV, highlighting lineage-specific performance and those that perform well universally despite emerging MPXV mutations. These 18 PCR primer sets included both qPCR and conventional PCR, which are both utilized in clinical settings. The conventional PCR method is not common in clinical testing, but allows Sanger Sequencing of the product. This conventional PCR approach is a crucial part of pathogen confirmation in the earlier stages of pathogen detection [35].

Additionally, we analyzed nucleotide conservation across all 5210 MPXV genomes and identified 173 conserved regions longer than 150 bp that could be used for future primer design. Together, these results provide guidance for MPXV PCR-based tests and show that the continuous monitoring of their performance is essential.

## 2. Materials and Methods

### 2.1. Primer and Probe Information

The details of the fourteen qPCR and four conventional PCR primers/probes are summarized in Table 1. The design of these primer sets was based on previous publications. In Table 1, the reported primer names were used, and the first author’s initial was appended in cases of duplicate primer names. The primer sets included E9L [17], B6R [17], G2R_G [18], G2R_WA [18], C3L [18], VEC [19], O2L [20], F3L_K [21], B2R [22], OPV [23], F3L_K [24], N3R [24], OPX [25], A4L [26], A39R [35], B2R [27], ATI [28], and HA [36] (Table 1). G2R_G, G2R_WA, and C3L are the WHO-recommended primer designs [37] for detecting both Clade I and II, and Clade II and Clade I, respectively; G2R_G also is the CDC-recommended [38] primer set.

### 2.2. MPXV Genome Extraction and Lineage Assignments

**Figure 1:**
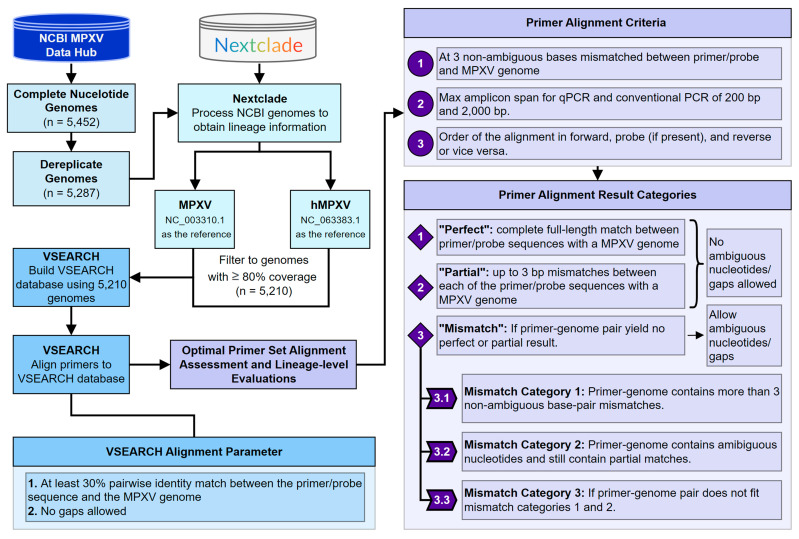
Detailed workflow describing the MPXV genome collection, lineage analysis, and MPXV primer evaluation. The MPXV genomes were downloaded from the NCBI viral genome resource, dereplicated, and analyzed using Nextclade software to determine the lineage and coverage information. A total of 80% coverage-filtered genomes were then used to build a database to which the MPXV primers were aligned and evaluated. The VSEARCH criteria and primer alignment criteria were used to select acceptable primer-genome pairs. The alignment results were then divided into “perfect”, “partial”, and “mismatch” categories based on the alignment quality. The “mismatch” category was further divided into three categories based on the VSEARCH results.

The workflow for the MPXV genome extraction and lineage assignments is summarized in Figure 1. A total of 5452 MPXV genomes were extracted from the NCBI viral genomes resource [34] on 21 May 2023, and the genomes were filtered according to monkeypox taxid (10244) and a minimum genome length of 150,000 bp. These genomes were then dereplicated, removing 164 duplicates and yielding 5287 unique genomes. Next, the genome lineages and coverage information were determined using Nextclade CLI [15] with hMPXV (latest updated date: 26 January 2023 with NC_063383.1 as the reference genome) and MPXV (latest updated date: 26 January 2023 with NC_003310.1 as the reference genome). Nextclade hMPXV provides refined Clade IIb lineage assignments, which follow the general convention of descending from either lineage A or B, followed by a numerical value indicating sublineage. The Nextclade MPXV reference was used to assign the non-Clade IIb genomes and was named “Clade”, followed by categorizing them as I, II, IIa, or IIb. The genomes were then filtered using a minimum coverage threshold of 80%, yielding a final set of 5210 genomes with complete lineage and coverage information.

### 2.3. Primer Alignment Evaluations

The primer alignment and results evaluations are summarized in Figure 1. The primer alignment evaluations were performed using VSEARCH [39] (v.2.17.1) by aligning each of the primer/probe sequences against a custom VSEARCH database of 5210 genomes (Table 1). The VSEARCH alignment between the primer/probe sequences and reference sequences allowed at least 30% of matches without any gaps. The results were subsequently processed in R [40] (v.4.2.2), with the primer filtering criteria listed in Figure 1. For a primer set to have a “perfect” genome match, the forward primer, reverse primer, and probe alignments were all required to match the corresponding genome sequence completely with no mismatch or gap. “Partial” matches were designated when one or more alignments in the primer set had 1–3 bp mismatches per forward, reverse, and probe. All of the other primer-genome pairs that failed these criteria were considered “mismatches” in this analysis. These mismatch primer-genome pairs were further evaluated by relaxing rule 1 from the primer alignment criteria (Figure 1) to include the primer binding results with more than three non-ambiguous nucleotide matches and alignment with ambiguous nucleotides. To avoid accepting primer binding from the wrong region of the MPXV genome, the search was narrowed to 3000 bp around the median of the forward and reverse start positions across all the perfect and partial alignments. These newly found primer-genome pairs were also required to conform to the two other primer alignment criteria. In addition, the multiple sequence alignment of 5210 MPXV genomes from Nextalign [14] (using the Clade II reference genome) was used to examine the presence of gaps or ambiguous nucleotides at the binding sites of these primers.

### 2.4. Inverted Regions and Low Mappability Region Identification

To assess the effects of the known repeats in the MPXV RefSeq genomes, genomic inverted regions (IRs) were identified using a Palindrome analyzer [41] with the following settings: an IR size between 6 and 30 bp, a spacer size between 0 and 10 bp, and allowed mismatch between 0 and 1 bp. GenMap [42] was used to assess the uniqueness and repetition of the two MPXV RefSeq genomes independently with a k-mer size of 50 and up to 4 bp mismatches between each k-mer and other parts of the same MPXV genome.

### 2.5. Conserved Region Identification

A multiple sequence alignment of all 5210 MPXV genomes included in this study was generated using Nextalign [14] with NC_063383.1 as the reference genome. Consecutive segments of ≥150 bp in length with ≥99% overall position consensus and ≥80% lineage-specific consensus were extracted in R [40] (v.4.2.2) using a custom script. Nucleotides from the 99% overall nucleotide consensus were required to match those of the lineage-specific consensuses. Genomic variation was also analyzed using the normalized Shannon equitability index described by Li et al. [43], which uses a log with a base of 5 for a tailored assessment of the genomic variation, incorporating four different nucleotides and gaps.

## 3. Results

### 3.1. MPXV Genomic Dataset Overview

**Figure 2:**
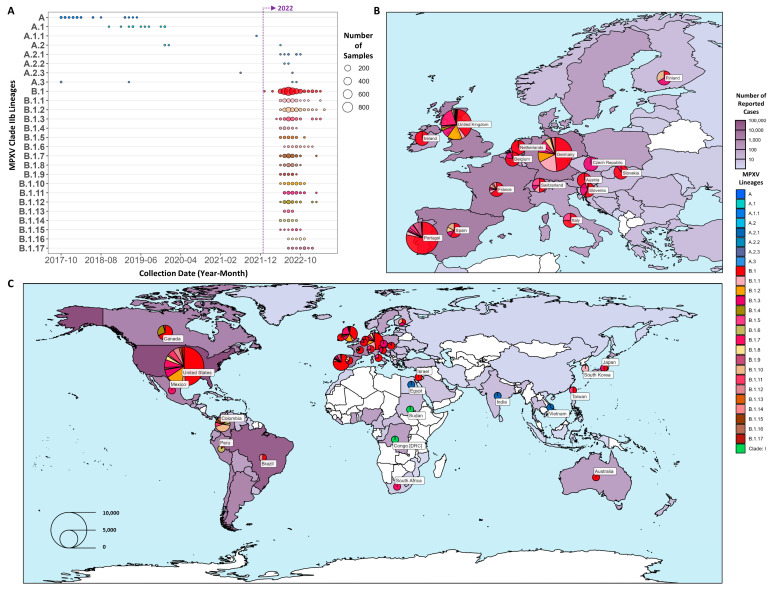
Summary of MPXV genome lineages, collection dates, and geolocations. (**A**) Collection date (aggregated by month) for all 26 Clade IIb sublineages. The purple vertical line indicates the beginning of 2022. European (**B**) and global (**C**) illustrations of lineage distributions across different regions, including all samples with an available sample collection time between 2022 and 2023. The regions with reported cases are shown in a purple gradient (white = low, dark purple = high) based on the cumulative number of Mpox cases as of June 2023 reported by the WHO. Each region with collected genomes also has colored pie charts representing the distribution of the MPXV lineages identified.

A set of 5210 MPXV genomes was curated from the NCBI viral genome resource [34] (Section 2), representing samples collected in 39 distinct regions of a time period from 1965 to 2023 (81 genomes were without sample collection dates). Of these genomes, 96.8% (5043 of 5210) were assigned to B lineages, while 1.7% (87 of 5210) were assigned to A lineages, all within Clade IIb (Figure 2A). The remaining 80 genomes (1.5%) were assigned to lineages outside Clade IIb, representing Clade I. One of these samples (KJ642617.1 collected in Nigeria in 1971 [44]) was classified as Clade IIb and did not belong to the A or B sublineages, suggesting that it was a distinct Clade IIb sublineage, consistent with its original reporting as Clade II [45]. This diversity of samples was observed across multiple continents with cases from the 2022–2023 period with 4819 samples, primarily North America (62.73%) and Europe (35.13%), and spanned regions where the number of cases in the 2022 Mpox outbreak was the highest (Figure 2B,C, Table S1).

### 3.2. *In silico* Evaluation of MPXV PCR-Based Assays

Using the database of the 5,210 curated MPXV genomes, we analyzed the alignment success of fourteen qPCR and four conventional PCR primer/probe designs, categorizing the results as “perfect”, “partial”, and “mismatch” across thirty lineages/clades (Section 2, Table 2, Table S2). Of the 18 primer sets analyzed, F3L_M, E9L, HA, and G2R_WA performed the best, with 99.46%, 99.10%, 97.17%, and 96.20% perfect matches, respectively (Table 2). F3L_M yielded 5182 perfect alignments and 17 partial alignments, all due to a single nucleotide mismatch at the 17th position in the probe sequence (Figure S1). Among the 17 F3L_M partial alignments, 15 came from Clade IIa, whereas one came from Clade I, and another originated from B.1. E9L had 5163 perfect alignments with one partial alignment to an A.1 lineage, caused by a single nucleotide mismatch near the 3′ end of the forward primer (Figure S2). HA had 5029 perfect alignments with 29 mismatches originating from different locations on the forward or reverse primers (Figure S3). G2R_WA had 5012 perfect alignments with two partial alignments to the B.1 lineages, all due to a single nucleotide mismatch near the 5′ end of the probe (Figure S4). Among these four primer sets, only HA and G2R_WA showed predominant mismatches against 26 Clade IIa genomes, while G2R_WA also displayed a high number of mismatches for certain B lineages (further details are provided in a subsequent section). Lastly, C3L (which targets Clade I only) had perfect alignment to 98.1% (51 of 52) of the Clade I genomes, with the remaining genome in the mismatched category.

The primer sets G2R_G, B6R, B7R, O2L, F3L_K, B2R_S, OPV, B2R_R, A4L, OPX, N3R, and ATI resulted in >90% partial matches among the 5210 genomes. G2R_G (the generic MPXV primer set) had 95.49% partial alignment, which was primarily caused by a single nucleotide mismatch in both the forward and reverse primers (Figure 3, Figure S5). Both G2R_G and OPX had two prevalent mismatches, while the others had a single prevalent mismatch with their primer and probe sequences. The detailed breakdown of the partial matches across the lineages is documented in Table S2 and Figures S1–S18. 

**Figure 3:**
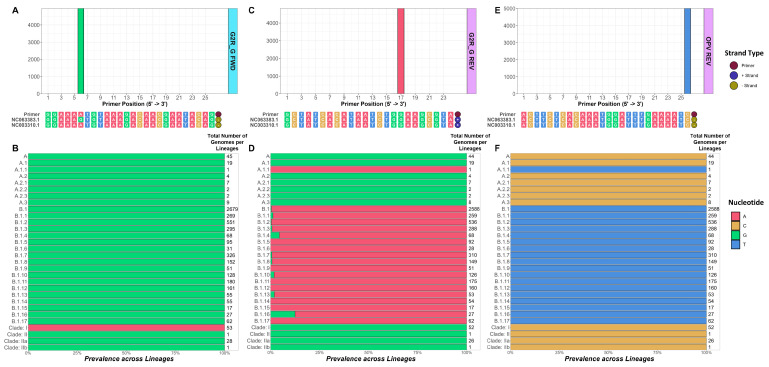
Single nucleotide mismatch patterns for the selected primers with respect to the RefSeq MPXV Clade I (NC_003310.1) and Clade II (NC_063383.1) reference genomes with more detailed clade/lineage-specific mismatches to the 5210 NCBI MPXV genomes analyzed. The G2R_G forward primer, G2R_G reverse primer, and OPV reverse primer are highlighted in panels (**A**,**B**), (**C**,**D**), and (**E**,**F**), respectively. Panels (**A**,**C**,**E**) detail the primer alignments to the reference genomes with the number of mismatched NCBI MPXV genomes shown at each primer position above, colored according to the nucleotide polymorphism. Panels (**B**,**D**,**F**) depict the fraction of genomes for each clade/lineage that harbors the mismatches highlighted in (**A**,**C**,**E**). In all panels, the nucleotides are colored as follows: A = red, C = yellow, G = green, T = blue. The strands of the Clade reference genome aligned sequences are indicated to the right of the sequence as follows: positive (+) strand = blue, negative (-) strand = yellow.

The primer sets analyzed in this study employed different approaches to validate their primer and probe sequences, typically using one or a few MPXV genomes (non-lineage specific). This approach limits the ability to check the primer/probe alignment sequence identity across the full repertoire of MPXV lineages or clades. Moreover, with the known elevated mutation rate for the MPXV genomes, we posited that mutations from specific, recently emerged lineages might be the reason many of the primers have 1 or 2 mismatches with the MPXV genomes analyzed (Table 2). We further investigated the exact sources of primer-genome mismatches by visualizing the Clade I and Clade II reference genome alignments as well as those of the various lineages/clades analyzed (Figure 3). The analysis of the G2R_G forward primer revealed that the single mismatches we detected were only with the Clade II reference genome but not with Clade I (Figure 3A,B). The analysis of the G2R_G reverse primer (Figure 3C,D) and the OPV reverse primer (Figure 3E,F) interestingly showed perfect alignment with both the Clade I and II reference genomes and instead revealed their mismatch occurred with A.1.1 and all B lineages. These observations highlight the importance of examining the binding of primers against the genomes representing all known lineages, as reference genomes are not always sufficient for the in silico assessment of PCR designs.

Another important consideration for PCR assay design is the length of the targeted amplicon region, which is known to impact the success and efficiency of PCR reactions through insertions or deletions near or at the primer binding sites. We, therefore, investigated the amplicon sizes to confirm the stability of each primer design, as our analyses accept any viable alignment less than 200 bp. The results indicated no amplicon length variations for 11 of the 14 qPCR primer sets across all perfect and partial alignments. The remaining qPCR primers, B7R, N3R, and O2L, showed slight length variations for a small number of partially matched genomes with deviations less than 6 bp (Figure S19). The amplicon length variations for conventional PCR had larger amplicon variations, except for the 70 bp A39R PCR product size. ATI generated amplicon sizes of 1000 bp and 1500 bp for the Clade I and Clade II MPXV genomes, respectively, and individual amplicon lengths varied by approximately 50 bp. Both B2R_R and HA showed varied amplicon lengths of more than 50 bp from <10 genomes. Together, these results show the stability of the amplicons for both qPCR and conventional PCR detections of perfect and partial primer-genome pairs.

### 3.3. Mismatch Primer-Genome Classification

To further assess the reason for primer-genome mismatches, we closely investigated the genomic context of the mismatches (Table 2). To our surprise, relaxing the 3 bp-level mismatches per primer sequence recovered a few (18 or less) additional pairs from each of our 18 primer sets (Table 2). The analysis of the genomic nucleotide compositions revealed that 91.86% of the 5210 MPXV genomes we analyzed contained fewer than 5% ambiguous nucleotides, indicating many genomes could have ambiguous nucleotides near or within the primer binding sites. Therefore, we further expanded our primer-genome alignment search to include alignments with ambiguous nucleotides at the potential primer binding sites, which were extrapolated from all the perfectly and partially matched primer binding positions (Figure S20 and Section 2). The results divided these misalignments into two categories: the presence of ambiguous nucleotides (i.e., “N”) or missing acceptable alignments (Table 2).

**Figure 4:**
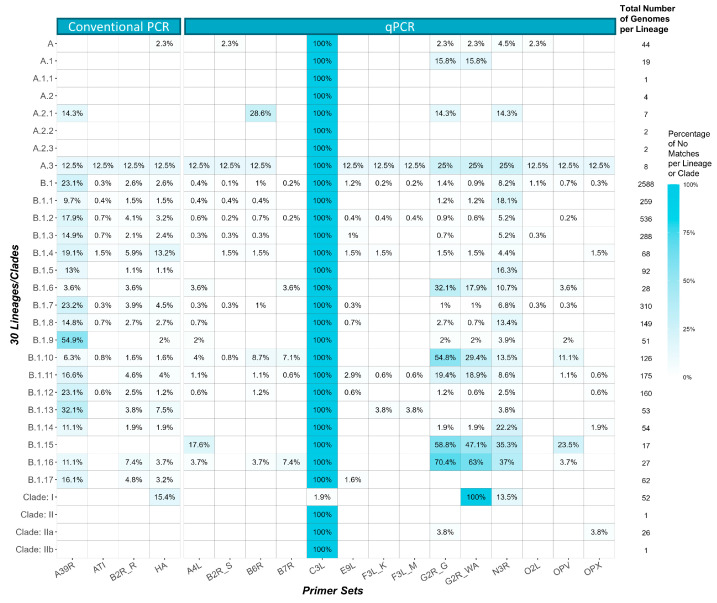
Percentage of mismatches per lineage for each of the primer sets. The total number of genomes per lineage is displayed on the right. All blank cells represent the primer alignments in either the perfect or partial match category (i.e., 0% mismatches).

Next, we fully characterized the relationship between the MPXV clades/lineages and the “mismatch” primer-genome pairs, revealing that most mismatches were randomly distributed across MPXV clades/lineages with <1% mismatches (Figure 4). However, we observed a high mismatch prevalence (>25%) between certain B lineages and A39R, G2R_G, G2R_WA, N3R, and OPV primers (Figure 4). Both G2R_WA and G2R_G showed an elevated (10% to 70%) presence of ambiguous nucleotides, as well as 10–20% of gaps for some of the B lineages (Figure S21). Similarly, N3R displayed 10–37% of mismatches against B.1.16 and B.1.17, with an increased prevalence of ambiguous nucleotides for the N3R reverse primer binding region (Figure S22). We also noticed an increase (~20%) of gaps that covered 75% of the N3R amplicon region with a total span of 150 bp, likely due to deletions of part of the N3R gene for certain B lineages. Moreover, A39R from the conventional PCR category also displayed a relatively high percentage of 25–50% of ambiguous nucleotides for most of the Clade IIb B lineages (Figure S23). Overall, these ambiguous nucleotides and gaps were identified across multiple lineages and genomes, suggesting these genomic regions are prone to deletion, or are difficult to sequence and may not be suitable for the in silico investigation of PCR assays designed to target them.

There are two possible reasons for the presence of poorly sequenced and assembled regions. Firstly, there is a large (~6.5 kb) inverted terminal region (ITR) located at the 5′ and 3′ ends of the MPXV genome with reported repetitive low complexity repeats [46,47] and deletions [48]. This large ITR includes both copies of the OPG022 gene, where both G2R_WA and G2R_G are targeted. Secondly, we found that the presence of inverted repeats (IRs) is prominent across the MPXV genomes, identifying 8930 and 9014 IRs for Clade I (NC_003310.1) and Clade II (NC_063383.1), respectively (Tables S3 and S4). We also investigated these reference genomes for repeat regions that might interfere with sequencing, confirming those ITRs and revealing a few short, low mappability regions across other parts of both MPXV genomics regions (Tables S5 and S6). The effect of these features at the beginning and end of the MPXV genomes could interfere with the sequencing and assemblies of these genomes, leading to the high prevalence of ambiguous nucleotides and gaps we observed. Since these genomes were independently sequenced and submitted to NCBI, it is unlikely these consistently poorly sequenced/assembled regions were due to randomness.

Lastly, all primer sets except B7R failed to detect a singular A.3 genome entry (FV537349.1), which is a modified microbial nucleic acid record that contains 32,593 substitutions and 3176 deletions against NC_063883.1, and this modified genome is not representative for the A.3 lineages in nature.

### 3.4. Consensus Region Identification

**Figure 5:**
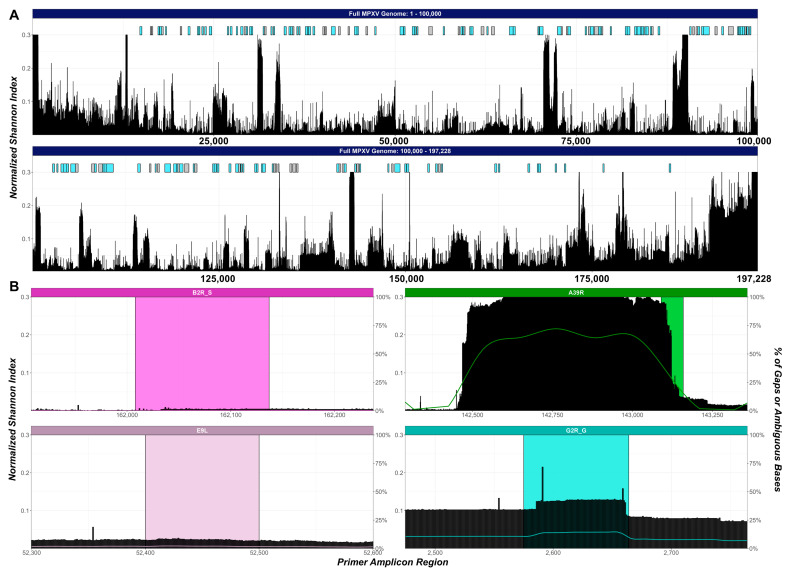
Consensus plot of the conserved regions and select primer sites across the MPXV genome. (**A**) Consensus plots with the sites of conserved regions across the entire MPXV genome. Conserved regions are colored blue if they overlap with an OPG gene; otherwise, the region is gray. (**B**) Consensus plots for the primer sets A39R, G2R_G, B2R_S, and E9L. The colored line represents the LOESS smoothing of the percentage of the gap or ambiguous base at each base position. The normalized Shannon index (black bar) is capped at 0.3 for display purposes. The select regions for the primer sets were 100 bp around the primer binding location except for A39R, which included 800 bp and 200 bp before and after the primer binding location.

In previous sections, we demonstrated that some primer binding regions contained gaps or ambiguous bases across different lineages, which interfered with in silico primer analyses. Here, we provide a more in-depth guide for future primer design and evaluation by identifying conserved regions across the MPXV genome using multiple alignment results from the 5210 genomes analyzed in this study (Section 2). Overall, we identified 173 conserved regions with a minimal length of 150 bp across the MPXV genome (mean length: 275bp, max length: 952 bp) (Table S7), and 115 of these were located within annotated OPG genes (Figure 5A). These segments contain ≥99% overall sequence consensus and ≥80% lineage-specific consensus to ensure the recorded nucleotides represent the population and lineage-specific major alleles.

Among these conserved regions identified, two overlapped with primers evaluated in this study (B2R_S and E9L, Figure 5B). The design for B2R_S was based on a hypothetical protein record from the Vaccinia virus (YP_233066.1), which resulted in a single nucleotide mismatch at the 18th position on the probe sequence across almost all 5210 genomes. If the correct nucleotide were used, one would obtain a highly conserved primer across almost all lineages. The E9L primer region also coincided with a conserved region, with a small increase in the normalized Shannon index due to the slight presence (~1–2%) of ambiguous nucleotides in this region (Figure 5B). On the other hand, the A39R forward primer overlapped with a poorly conserved region (>0.2 normalized Shannon index covering the forward primer binding sites), explaining why many of the alignments were in the mismatch categories (Table 2, Figure 5B). Similarly, G2R_G was found within a relatively poor conserved region (normalized Shannon index ~0.1) with two noticeable increases in the Shannon index corresponding to the two commonly mismatched nucleotides in the forward and reverse primers (Figure 3 and Figure 5B). Together, these results reveal multiple poorly conserved regions that were independently sequenced, which indicates that certain MXPV regions cannot be effectively surveilled in silico as novel MXPV lineages emerge and should, therefore be avoided in PCR designs.

## 4. Discussion

The rapid development of accurate and robust detection assays is crucial for the control and monitoring of public health emergencies. With the advancement of various sequencing methods, it has become more affordable to assemble new genomes for continuous monitoring of genomic evolutions of pathogens. This allowed us to monitor primer detection assay binding specificities toward all available genomes to evaluate primer binding with specific MPXV lineages in silico. While perfect alignment might not be required for the accurate detection of pathogens with high viral loads, a previous study showed that an MPXV detection assay returned negative results from Mpox patients from saliva, nasopharyngeal, urine, semen, and feces samples, where the viral loads might be much lower than lesion swabs [49]. Another study investigated the difference in the limit of detection (LoD) of G2R_G with the original (two mismatches) and custom (no mismatches) primer sets that resulted in 95% LoD of 10.8 copies per reaction and 2.7 copies per reaction, respectively [31]. Together, to accurately maximize the detection capability of MPXV detection assays on different sample types and different viral loads, it is crucial that primer designs retain perfect matches against MPXV genomes and lineages.

In this study, we evaluated 18 primer sets and their performance across 5210 MPXV genomes and 30 different lineages to highlight the need for constant monitoring of emerging mutations and their potential impacts on PCR-based MPXV diagnostic testing. Our results showed the E9L, F3L_M, C3L, HA, and G2R_WA primer sets were the top performers based on their total amount of perfect alignments against their designed MPXV lineages. When we allowed up to three mismatches per forward, reverse, or probe (if present) sequence, all of the primer sets except A39R could identify more than 91% of the 5120 genomes with partial or perfect alignments, with almost all alignments with at most two nucleotide mismatches in total. Almost all these mismatches were prevalent among the 2022 Mpox outbreak genomes under the Clade IIb A and B lineages, with only I7L detected in most of the A lineages. This highlights the urgent need to continuously monitor newer MPXV genomes, as these mutations were likely not present in the MPXV genome templates used when designing the MPXV detection assays.

When we investigated the mismatched primer-genome pairs, we discovered many lineages consisting of poorly sequenced and assembled regions containing 10% to 70% of ambiguous nucleotides. These regions were identified on different MPXV lineages from different samples collected at different places and times and are thus a likely reflection of the low complexity or repetitive MPXV genomic regions that interfered with the sequencing. Because MPXV genomes are brimming with inverted regions, their interference with different sequencing methods required further investigation to determine the optimal and consistent sequencing method of the MPXV genome. Moreover, the presence of up to 20% gaps at primer binding sites for G2R_WA, G2R_G, and N3R matched previously reported deletions of the crmB [48] and N3R [50] genes. These primer targets with known deletions should be avoided for further primer development.

Our examination of the conserved regions across MPXV genomes confirms the optimal performance of the E9L primer set. We also identified 173 conserved regions with no lineage biases within both MPXV reference genomes. However, IRs still permeate these regions, and because these IR regions have higher mutation rates [12], special attention is still needed for nucleotide polymorphism investigations. In general, repetitive regions have greater genome instability and are prone to structural variants (SVs) such as insertions and deletions.

In conclusion, our study demonstrated the critical need for conducting the lineage-specific examination of MPXV primer sets and continuously evaluating primer-genome binding efficiency to ensure the recommended primer sets are optimal against all MPXV lineages and clades. Our work developed a framework for conducting lineage-specific evaluations of primers against large genome databases. This framework could be used to enhance the monitoring of public health emergencies by providing an accurate assessment of the available detection assays.

## Figures and Tables

**Table 1:** Conventional and qPCR-based primer sets designed to target MPXV. Each primer set is provided on a single line showing the primer name and corresponding gene target, Orthopoxvirus gene (OPG) ID, primer/probe length, GC content, and target amplicon length.

Primer Name; Target Gene Name	Orthopoxvirus Genes (OPG)	Primer Length			GC Content ^†^	Amplicon Length *
		Forward	Reverse	Probe		
E9L; DNA polymerase	OPG071	23	28	32	37.25%	101
B6R; EEV type-I membrane glycoprotein	OPG190	28	23	14	37.25%	83
G2R_G; Crm-B secreted TNF-alpha-receptor-like protein	OPG002	26	24	30	38.00%	90
G2R_WA; Crm-B secreted TNF-alpha-receptor-like protein	OPG002	20	23	26	46.51%	82/85
C3L; Complement control protein	OPG032	24	24	30	39.58%	100/N/A
B7R; Ankyrin-like protein	OPG191	22	23	28	40.00%	99
O2L; NFkB inhibitor	OPG038	20	22	25	50.00%	96
F3L_M; Double-stranded RNA binding protein	OPG065	25	23	25	43.75%	79
B2R_S; Schlafen	OPG188	19	22	27	34.15%	130
OPV; Viral core cysteine proteinase	OPG083	24	26	20	36.00%	129
F3L_K; Double-stranded RNA binding protein	OPG065	22	21	20	48.84%	107/106
N3R; Brix domain protein	OPG016	26	25	21	37.25%	139
OPX; DNA polymerase	OPG071	22	26	29	33.33%	52/87
A4L; A5L protein-like	OPG130	19	18	29	59.46%	115/217
A39R; IEV transmembrane phosphoprotein	OPG164	22	19	N/A	48.78%	70
B2R_R; Schlafen	OPG188	17	17	N/A	38.24%	406
ATI; No overlapping gene	No overlapping	16	18	N/A	32.35%	1067/ 1545
HA; Hemagglutinin	OPG185	21	20	N/A	36.59%	1176/ 1175

^†^ GC content is based on the forward and reverse primers. * Single entry represents matching amplicon lengths between the Clade I reference strain and the Clade II reference strain. Two entries delimited by “/” are the Clade I reference strain followed by the Clade II reference strain. N/A represents no alignment for the corresponding strain. Primer sets without probe lengths (N/A) are conventional PCR designs.

**Table 2:** Primer alignment breakdown with mismatched positions and the categorization of the mismatched primer-genome pairs.

Primer	Perfect Alignment	Partial Alignments (≤3 bp Mismatches)	Predominant Partial Mismatch Position	Total Mismatches	Partial Alignment (>3 bp Forward, Reverse, or Probe Mismatches)	FWD or REV Alignment Containing Ambiguous Nucleotides	Missing Acceptable Alignments from Either FWD or REV	Missing Acceptable Alignments from PROBE
A39R	52	4135	19th position on the forward primer	1023	1	724	298	-
A4L	-	5178	1st position of the reverse primer	32	-	16	11	5
ATI	-	5190	1st position on the reverse primer	20	14	1	5	-
B2R_R	79	4987	14th position on the forward primer	144	2	79	63	-
B2R_S	-	5199	16th position of the probe	11	-	5	5	1
B6R	52	5104	1st position of the probe	54	-	18	34	2
B7R	78	5114	10th position of the probe	18	-	2	16	-
C3L	51	-	N/A	5159	-	47	5100	12
E9L	5163	1	N/A	46	-	13	26	7
F3L_K	51	5146	1st position of the reverse primer	13	1	7	4	1
F3L_M	5182	17	N/A	11	-	6	5	-
G2R_G	52	4951	6th position of the forward primer 17th position of the reverse primer	207	9	114	68	16
G2R_WA	5012	2	N/A	196	-	-	90	106
HA	5029	29	N/A	152	1	57	94	-
N3R	72	4695	12th position of the probe	443	-	72	367	4
O2L	52	5126	18th position of the probe	32	18	3	7	4
OPV	163	5003	26th position of the reverse primer	44	1	16	14	13
OPX	-	5196	11th position of the forward primer 12th position of the reverse primer	14	-	10	2	2

Cells with “-” represent zero. N/A represents no applicable information.

## Data Availability

The data used in the study were all extracted from the National Institute of Health Virus database.

## Supplementary Materials

The following supporting information can be downloaded at: https://zenodo.org/records/10049276, Figure S1–S18: Individual primer diagnostic plot for 18 primer sets; Figure S19: Amplicon size distribution across 18 primer sets; Figure S20: Distribution of the start position of the forward and reverse primers across 18 primer sets; Figure S21–S23: Detailed primer binding sites lineage-specific binding performance for G2R_WA/G2R_G, N3R, and A39R primer sets; Table S1: 5210 MPXV Genome Lineages Breakdown across Multiple Geological Locations. Table S2: Perfect, partial, and mismatch categories for each of the primer sets breakdown across 30 lineages/clades; Table S3: Inverted regions identified across the NC_068336.1 MPXV genome; Table S4: Inverted regions identified across the NC_003310.1 MPXV genome; Table S5: Low mappability regions across the NC_068336.1 MPXV genome; Table S6: Low mappability regions across NC_003310.1 MPXV genome; Table S7: 173 conserved regions identified based on the multiple genome alignments using NC_068831.1 as the reference. The genome start and stop locations are based on the location in NC_068336.1.
