# Supplementary material for: An In Silico Analysis of PCR-Based Monkeypox Virus Detection Assays: A Case Study for Ongoing Clinical Surveillance
Source: Viruses. 2023 Nov 27;15(12):2327. doi: 10.3390/v15122327 (PMC10747849; doi:10.3390/v15122327)
Supplement: Supplementary file 1 [file viruses-15-02327-s001.zip › MPXV_PrimerAnlaysis_SupplementaryFigures.pdf]

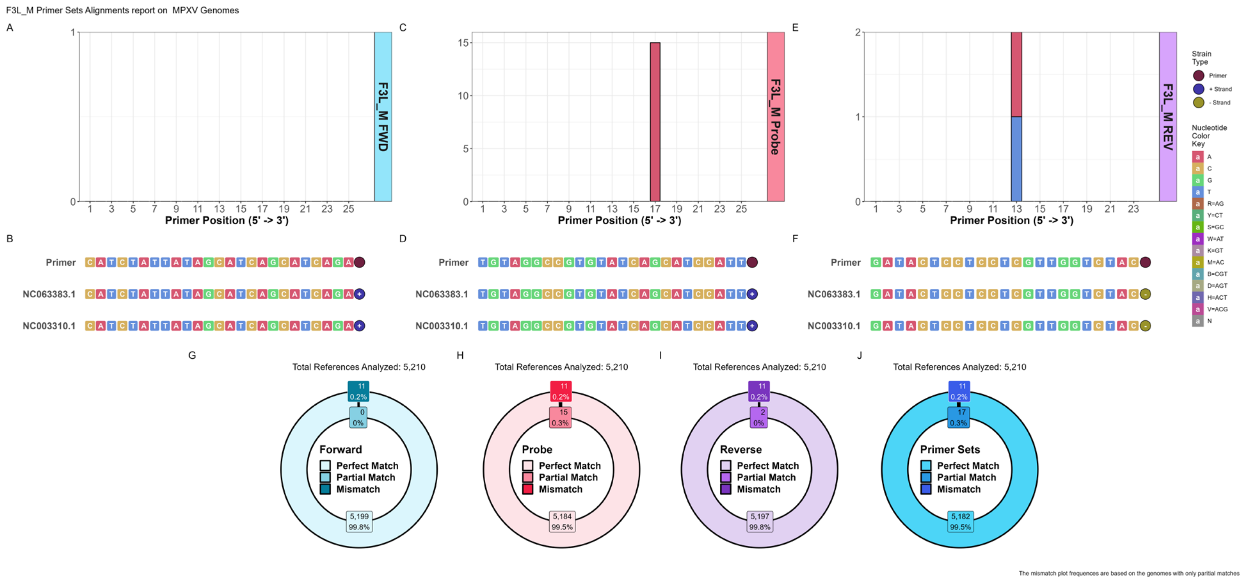

Supplementary Figure 1. The diagnostic plot of the forward, probe, and reverse primer alignment to 5,210 genomes of primer F3L_M. The partial alignment patterns across the forward, probe, and reverse sequences are highlighted in panels **A, C,** and **E,** respectively. The forward, reverse, and probe sequences that targeted the RefSeq MPXV Clade I (NC_003310.1) and Clade II (NC_063383.1) reference genome regions are illustrated in panels **B, D,** and **F,** respectively. Panels **G, H, I,** and **J** demonstrate the perfect, partial, and mismatch count and percentage for the forward, probe, reverse, and overall performance.

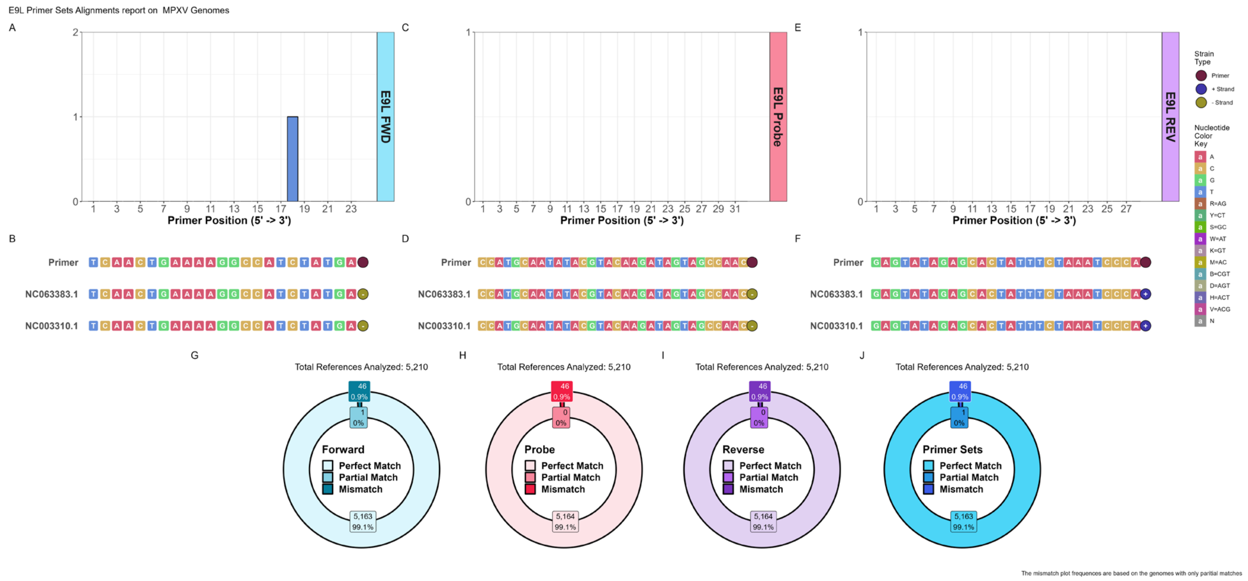

Supplementary Figure 2. The diagnostic plot of the forward, probe, and reverse primer alignment to 5,210 genomes of primer E9L. The partial alignment patterns across the forward, probe, and reverse sequences are highlighted in panels **A, C,** and **E,** respectively. The forward, reverse, and probe sequences that targeted the RefSeq MPXV Clade I (NC_003310.1) and Clade II (NC_063383.1) reference genome regions are illustrated in panels **B, D,** and **F,** respectively. Panels **G, H, I,** and **J** demonstrate the perfect, partial, and mismatch count and percentage for the forward, probe, reverse, and overall performance.

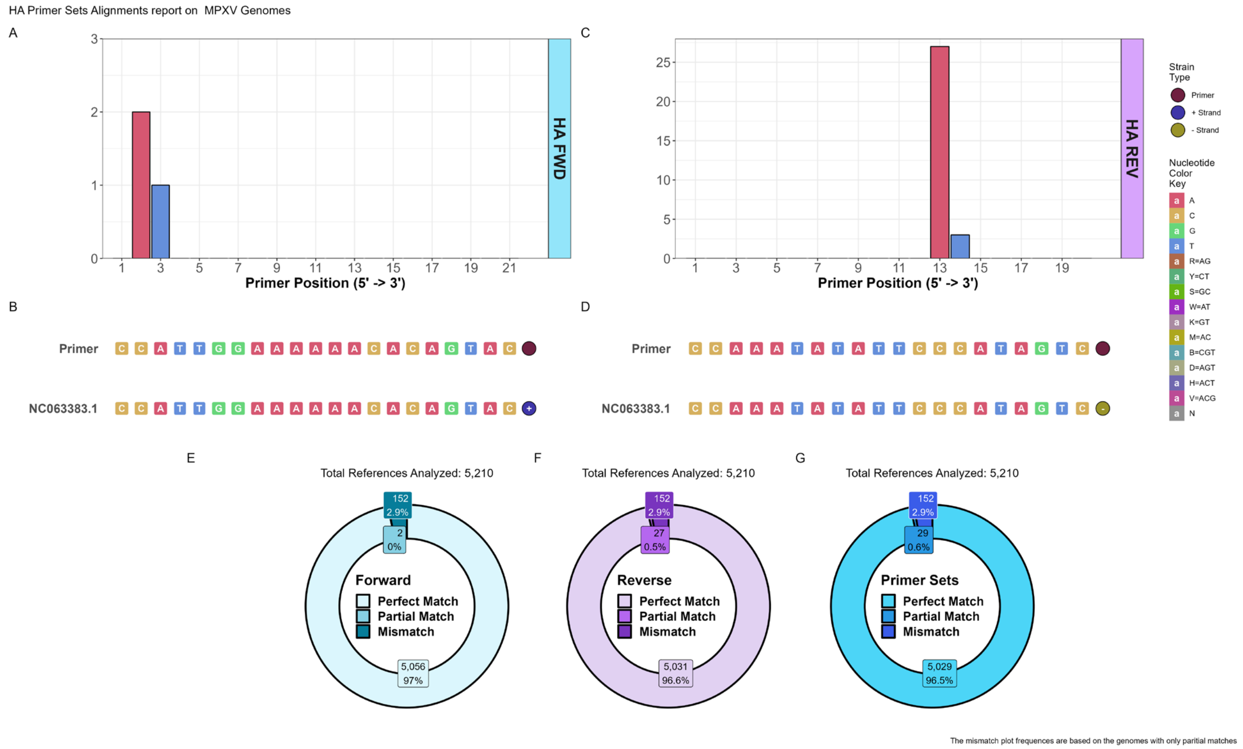

Supplementary Figure 3. The diagnostic plot of the forward and reverse primer alignment to 5,210 genomes of primer HA. The partial alignment patterns across the forward and reverse sequences are highlighted in panels **A** and **C,** respectively. The forward, reverse, and probe sequences that targeted the RefSeq MPXV Clade II (NC_063383.1) reference genome regions are illustrated in panels **B** and **D,** respectively. Panels **E, F,** and **G** demonstrate the perfect, partial, and mismatch count and percentage for the forward, reverse, and overall performance.

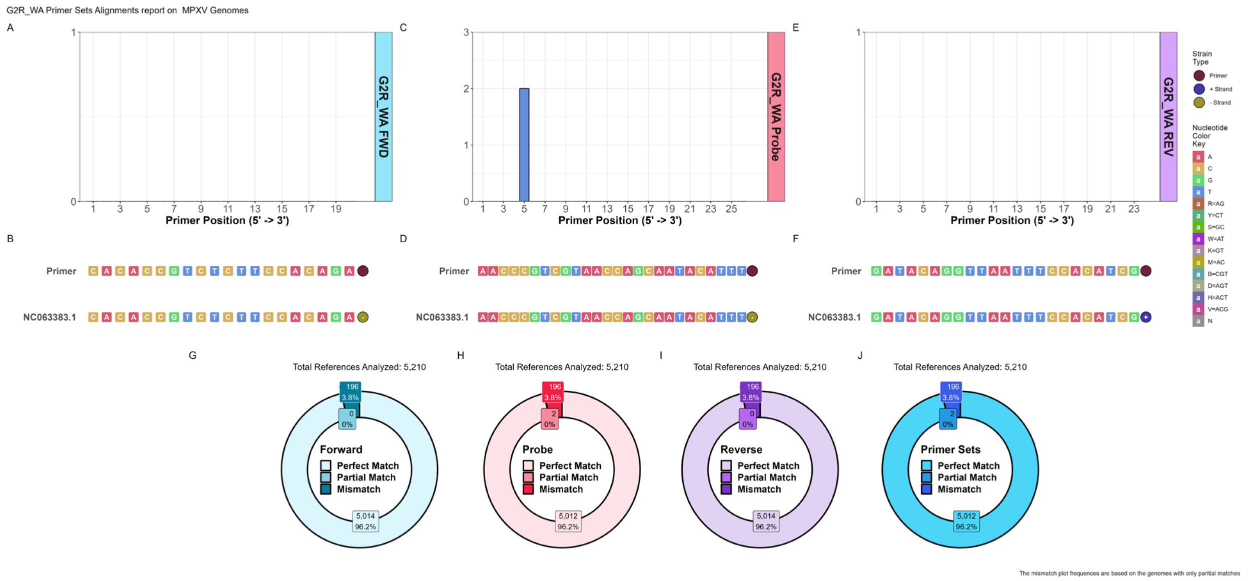

Supplementary Figure 4. The diagnostic plot of the forward, probe, and reverse primer alignment to 5,210 genomes of primer G2R_WA. The partial alignment patterns across the forward, probe, and reverse sequences are highlighted in panels **A, C,** and **E,** respectively. The forward, reverse, and probe sequences that targeted the RefSeq MPXV Clade I (NC_003310.1) and Clade II (NC_063383.1) reference genome regions are illustrated in panels **B, D,** and **F,** respectively. Panels **G, H, I,** and **J** demonstrate the perfect, partial, and mismatch count and percentage for the forward, probe, reverse, and overall performance.

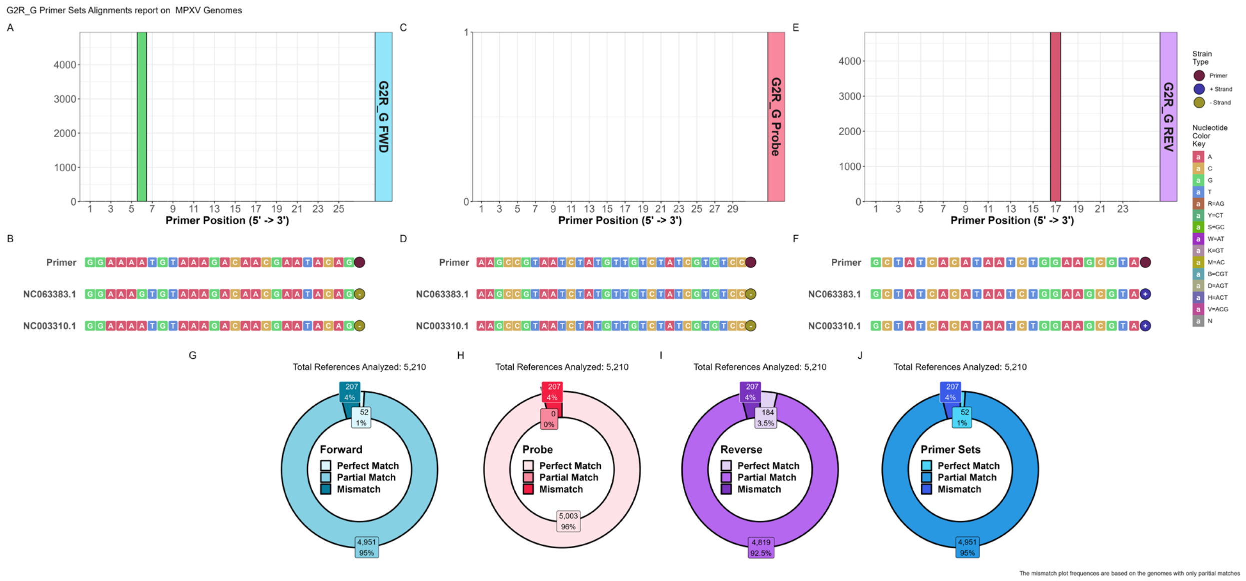

Supplementary Figure 5. The diagnostic plot of the forward, probe, and reverse primer alignment to 5,210 genomes of primer G2R_G. The partial alignment patterns across the forward, probe, and reverse sequences are highlighted in panels **A, C,** and **E,** respectively. The forward, reverse, and probe sequences that targeted the RefSeq MPXV Clade I (NC_003310.1) and Clade II (NC_063383.1) reference genome regions are illustrated in panels **B, D,** and **F,** respectively. Panels **G, H, I,** and **J** demonstrate the perfect, partial, and mismatch count and percentage for the forward, probe, reverse, and overall performance.

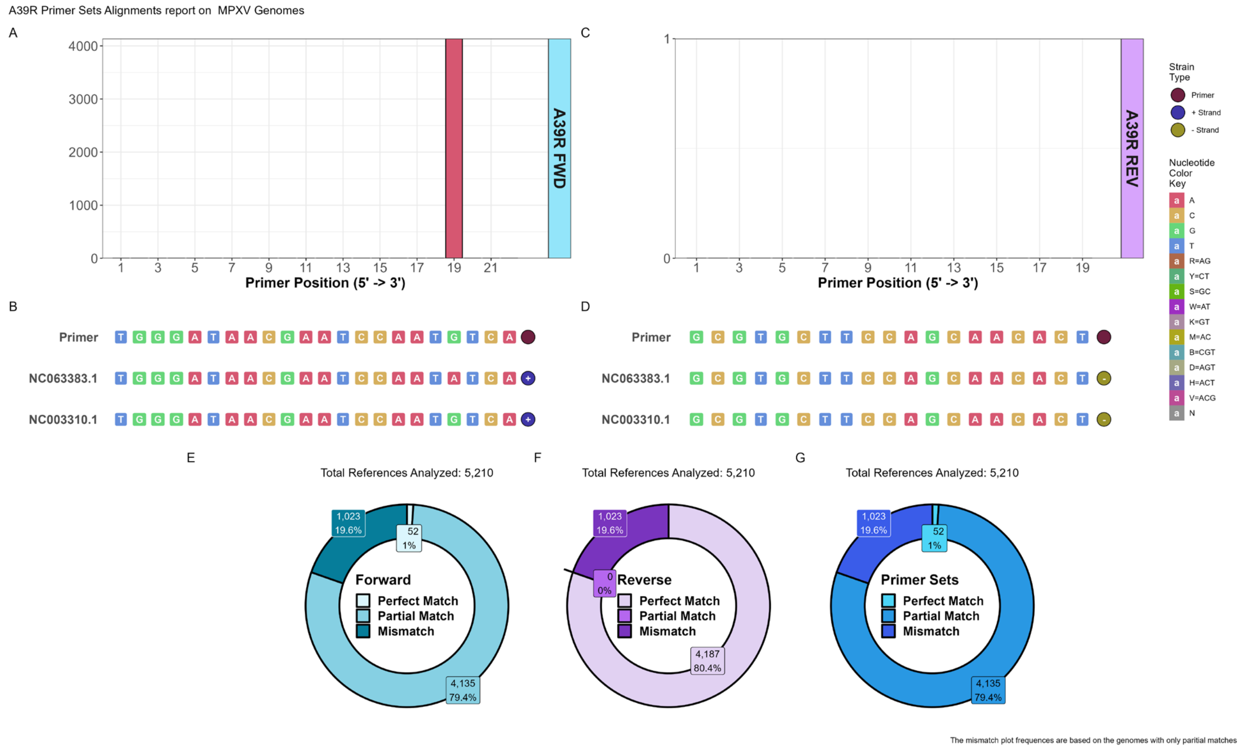

Supplementary Figure 6. The diagnostic plot of the forward and reverse primer alignment to 5,210 genomes of primer A39R. The partial alignment patterns across the forward and reverse sequences are highlighted in panels **A** and **C,** respectively. The forward, reverse, and probe sequences that targeted the RefSeq MPXV Clade I (NC_003310.1) and Clade II (NC_063383.1) reference genome regions are illustrated in panels **B** and **D,** respectively. Panels **E, F,** and **G** demonstrate the perfect, partial, and mismatch count and percentage for the forward, reverse, and overall performance.

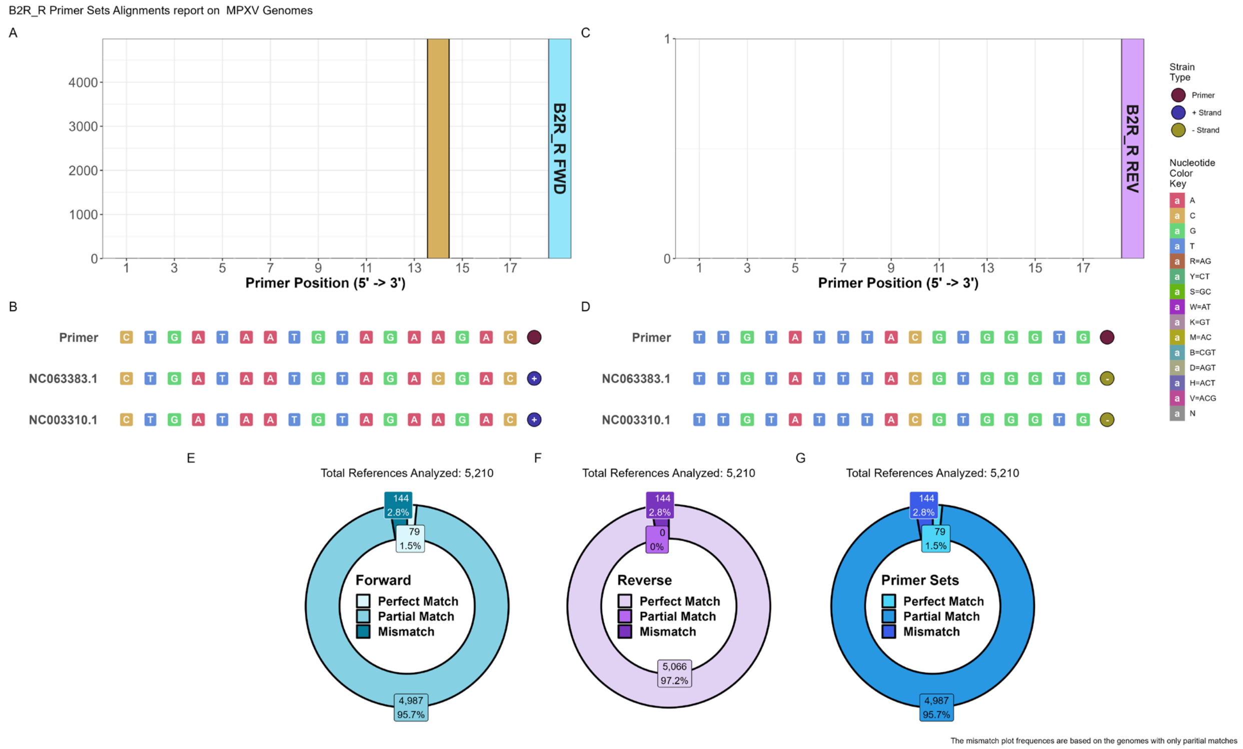

Supplementary Figure 7. The diagnostic plot of the forward and reverse primer alignment to 5,210 genomes of primer B2R_R. The partial alignment patterns across the forward and reverse sequences are highlighted in panels **A** and **C,** respectively. The forward, reverse, and probe sequences that targeted the RefSeq MPXV Clade I (NC_003310.1) and Clade II (NC_063383.1) reference genome regions are illustrated in panels **B** and **D,** respectively. Panels **E, F,** and **G** demonstrate the perfect, partial, and mismatch count and percentage for the forward, reverse, and overall performance.

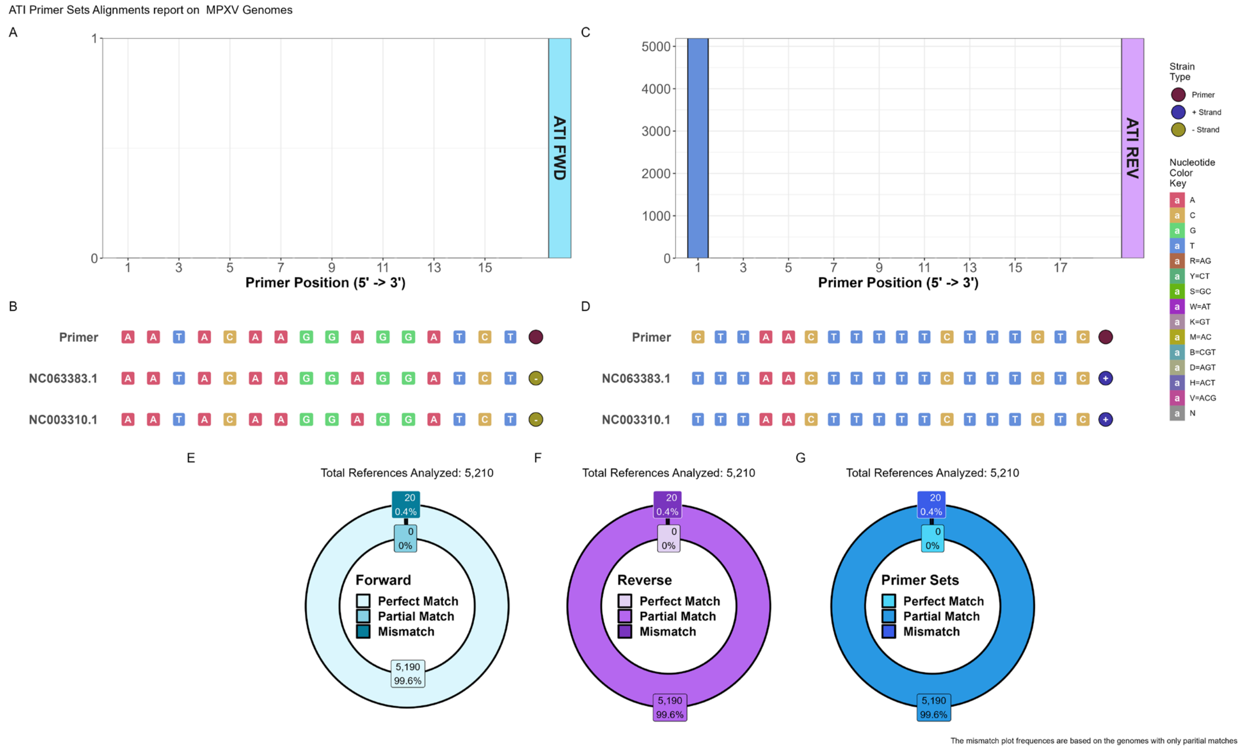

Supplementary Figure 8. The diagnostic plot of the forward and reverse primer alignment to 5,210 genomes of primer ATI. The partial alignment patterns across the forward and reverse sequences are highlighted in panels **A** and **C,** respectively. The forward, reverse, and probe sequences that targeted the RefSeq MPXV Clade I (NC_003310.1) and Clade II (NC_063383.1) reference genome regions are illustrated in panels **B** and **D,** respectively. Panels **E, F,** and **G** demonstrate the perfect, partial, and mismatch count and percentage for the forward, reverse, and overall performance.

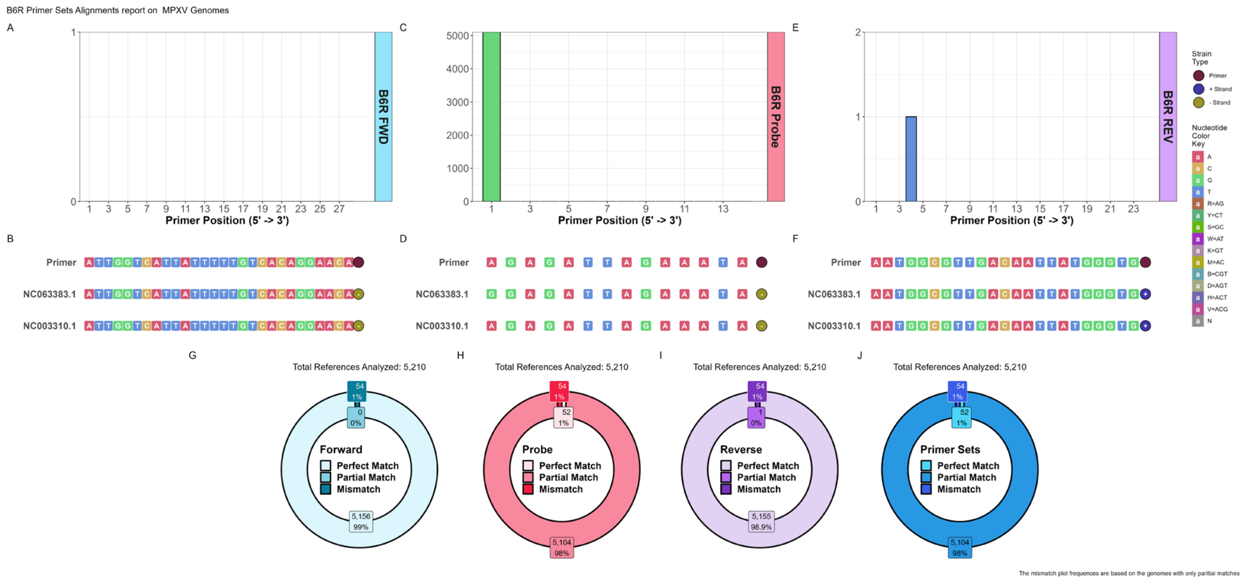

Supplementary Figure 9. The diagnostic plot of the forward, probe, and reverse primer alignment to 5,210 genomes of primer B6R. The partial alignment patterns across the forward, probe, and reverse sequences are highlighted in panels **A, C,** and **E,** respectively. The forward, reverse, and probe sequences that targeted the RefSeq MPXV Clade I (NC_003310.1) and Clade II (NC_063383.1) reference genome regions are illustrated in panels **B, D,** and **F,** respectively. Panels **G, H, I,** and **J** demonstrate the perfect, partial, and mismatch count and percentage for the forward, probe, reverse, and overall performance.

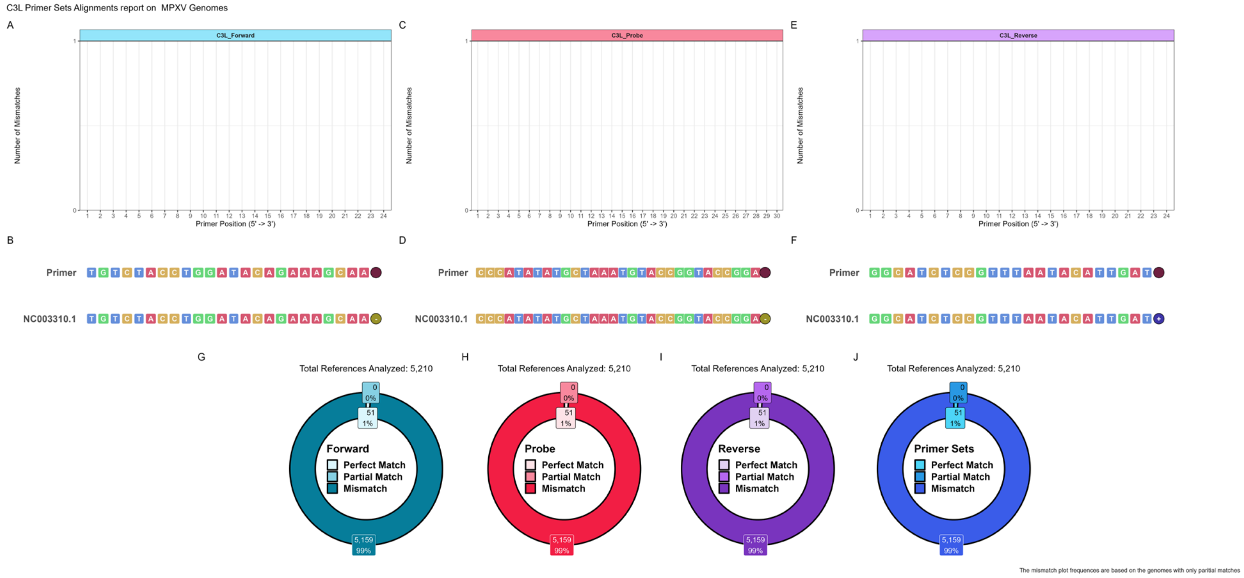

Supplementary Figure 10. The diagnostic plot of the forward, probe, and reverse primer alignment to 5,210 genomes of primer C3L. The partial alignment patterns across the forward, probe, and reverse sequences are highlighted in panels **A, C,** and **E,** respectively. The forward, reverse, and probe sequences that targeted the RefSeq MPXV Clade I (NC_003310.1) reference genome regions are illustrated in panels **B, D,** and **F,** respectively. Panels **G, H, I,** and **J** demonstrate the perfect, partial, and mismatch count and percentage for the forward, probe, reverse, and overall performance.

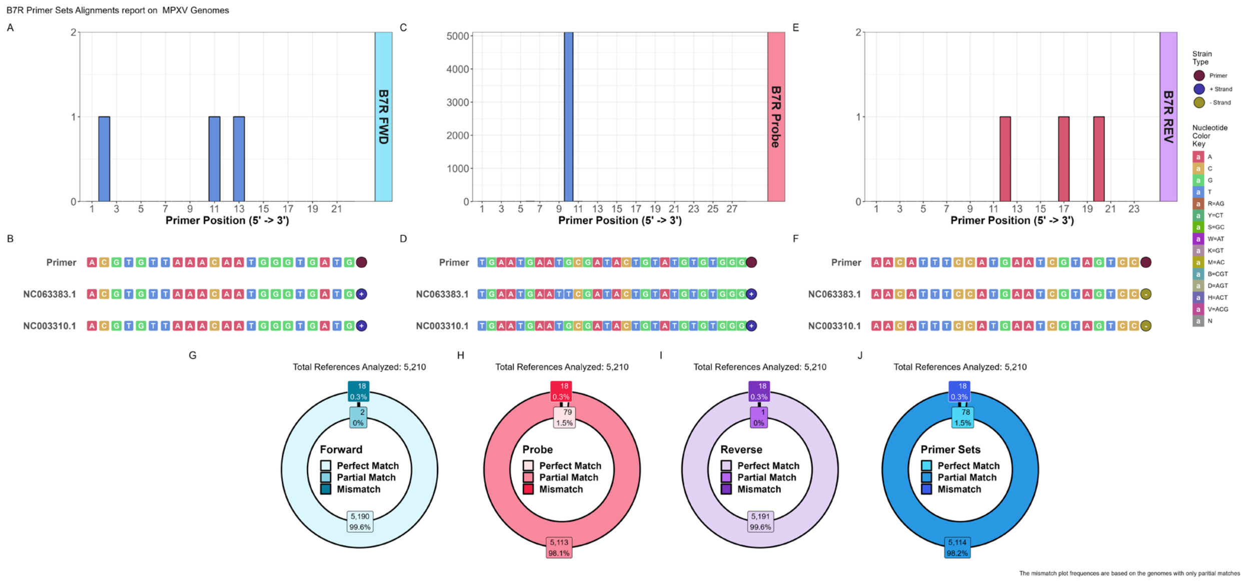

Supplementary Figure 11. The diagnostic plot of the forward, probe, and reverse primer alignment to 5,210 genomes of primer B7R. The partial alignment patterns across the forward, probe, and reverse sequences are highlighted in panels **A, C,** and **E,** respectively. The forward, reverse, and probe sequences that targeted the RefSeq MPXV Clade I (NC_003310.1) and Clade II (NC_063383.1) reference genome regions are illustrated in panels **B, D,** and **F,** respectively. Panels **G, H, I,** and **J** demonstrate the perfect, partial, and mismatch count and percentage for the forward, probe, reverse, and overall performance.

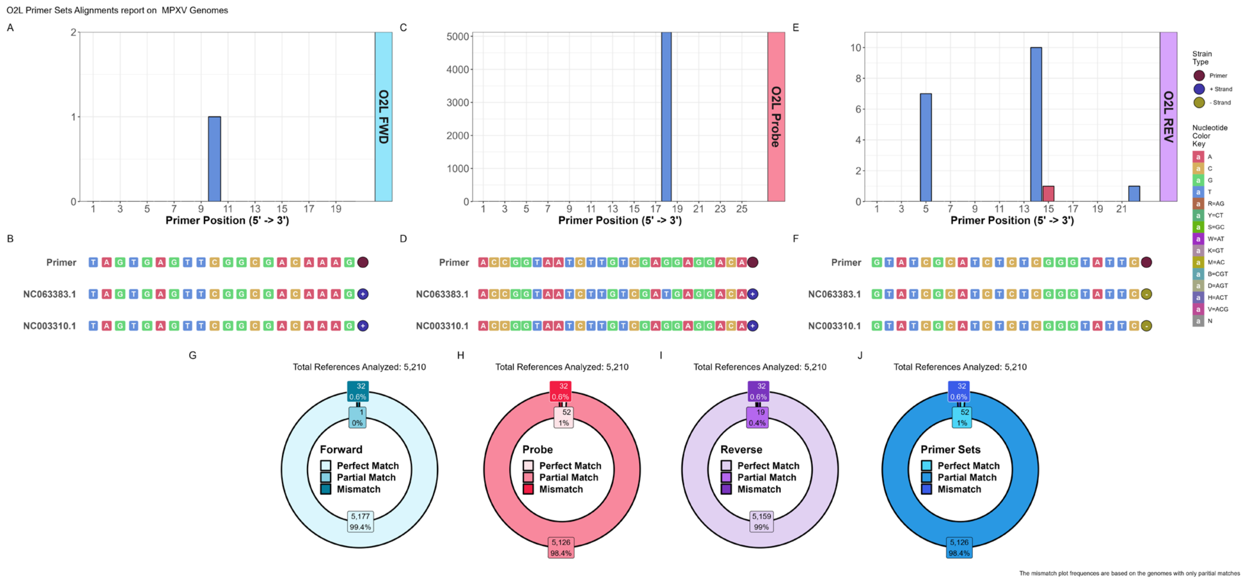

Supplementary Figure 12. The diagnostic plot of the forward, probe, and reverse primer alignment to 5,210 genomes of primer O2L. The partial alignment patterns across the forward, probe, and reverse sequences are highlighted in panels **A, C,** and **E,** respectively. The forward, reverse, and probe sequences that targeted the RefSeq MPXV Clade I (NC_003310.1) and Clade II (NC_063383.1) reference genome regions are illustrated in panels **B, D,** and **F,** respectively. Panels **G, H, I,** and **J** demonstrate the perfect, partial, and mismatch count and percentage for the forward, probe, reverse, and overall performance.

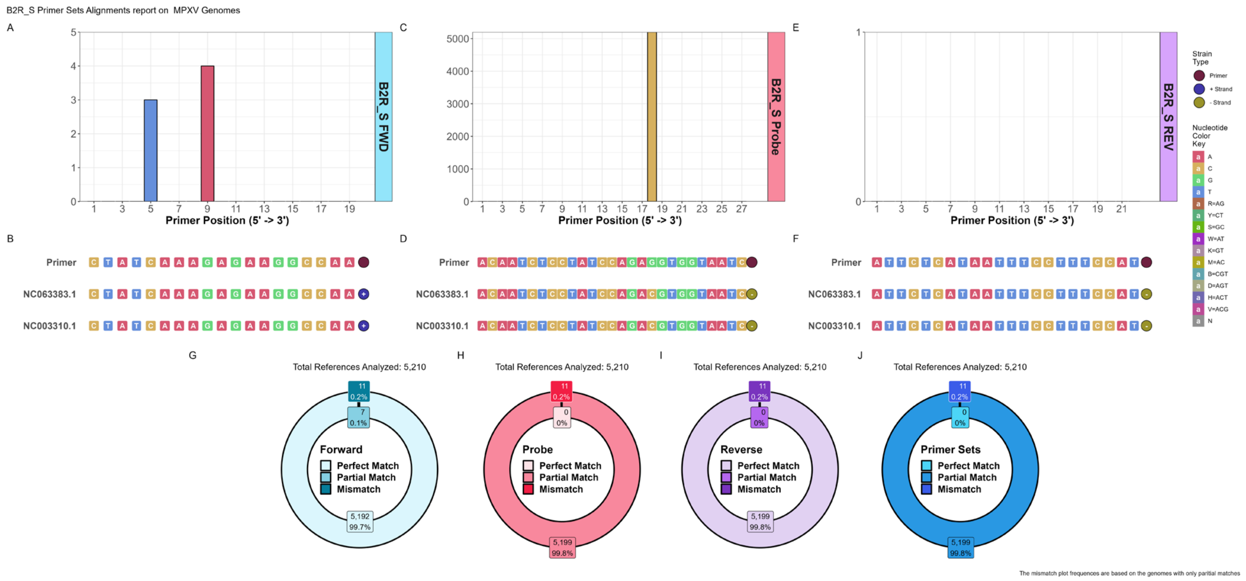

Supplementary Figure 13. The diagnostic plot of the forward, probe, and reverse primer alignment to 5,210 genomes of primer B2R_S. The partial alignment patterns across the forward, probe, and reverse sequences are highlighted in panels **A, C,** and **E,** respectively. The forward, reverse, and probe sequences that targeted the RefSeq MPXV Clade I (NC_003310.1) and Clade II (NC_063383.1) reference genome regions are illustrated in panels **B, D,** and **F,** respectively. Panels **G, H, I,** and **J** demonstrate the perfect, partial, and mismatch count and percentage for the forward, probe, reverse, and overall performance.

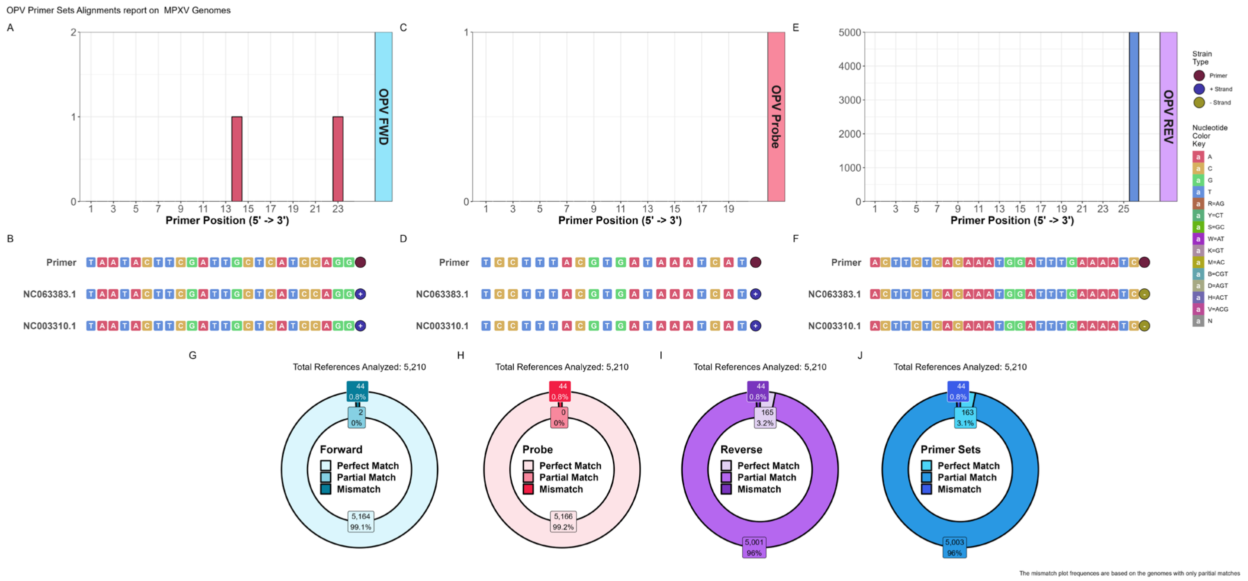

Supplementary Figure 14. The diagnostic plot of the forward, probe, and reverse primer alignment to 5,210 genomes of primer OPV. The partial alignment patterns across the forward, probe, and reverse sequences are highlighted in panels **A, C,** and **E,** respectively. The forward, reverse, and probe sequences that targeted the RefSeq MPXV Clade I (NC_003310.1) and Clade II (NC_063383.1) reference genome regions are illustrated in panels **B, D,** and **F,** respectively. Panels **G, H, I,** and **J** demonstrate the perfect, partial, and mismatch count and percentage for the forward, probe, reverse, and overall performance.

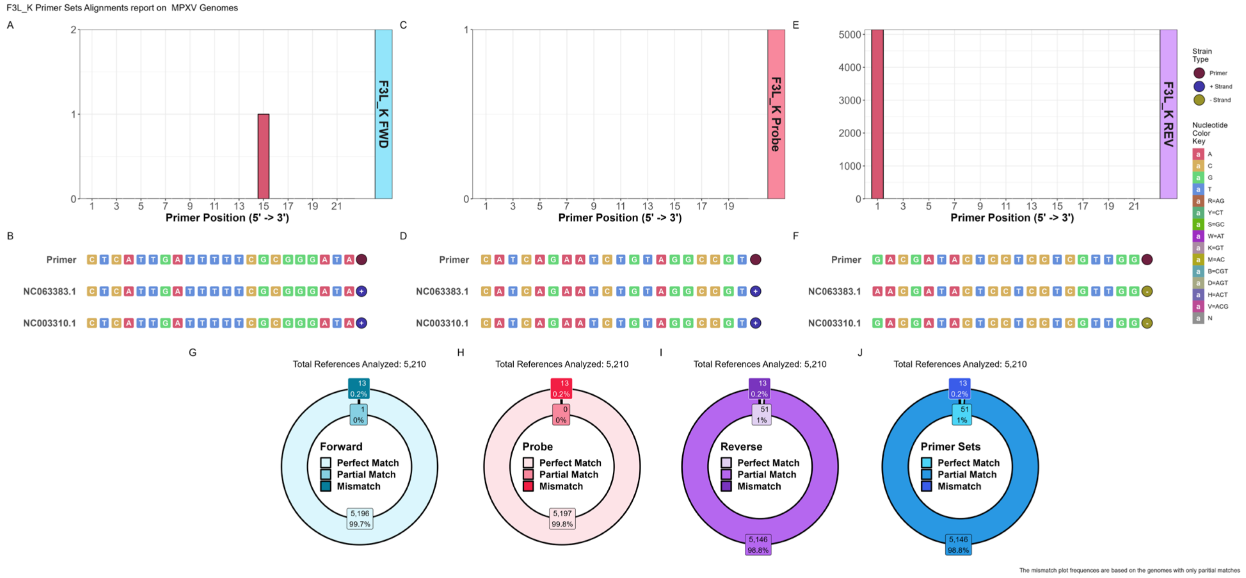

Supplementary Figure 15. The diagnostic plot of the forward, probe, and reverse primer alignment to 5,210 genomes of primer F3L_K. The partial alignment patterns across the forward, probe, and reverse sequences are highlighted in panels **A, C,** and **E,** respectively. The forward, reverse, and probe sequences that targeted the RefSeq MPXV Clade I (NC_003310.1) and Clade II (NC_063383.1) reference genome regions are illustrated in panels **B, D,** and **F,** respectively. Panels **G, H, I,** and **J** demonstrate the perfect, partial, and mismatch count and percentage for the forward, probe, reverse, and overall performance.

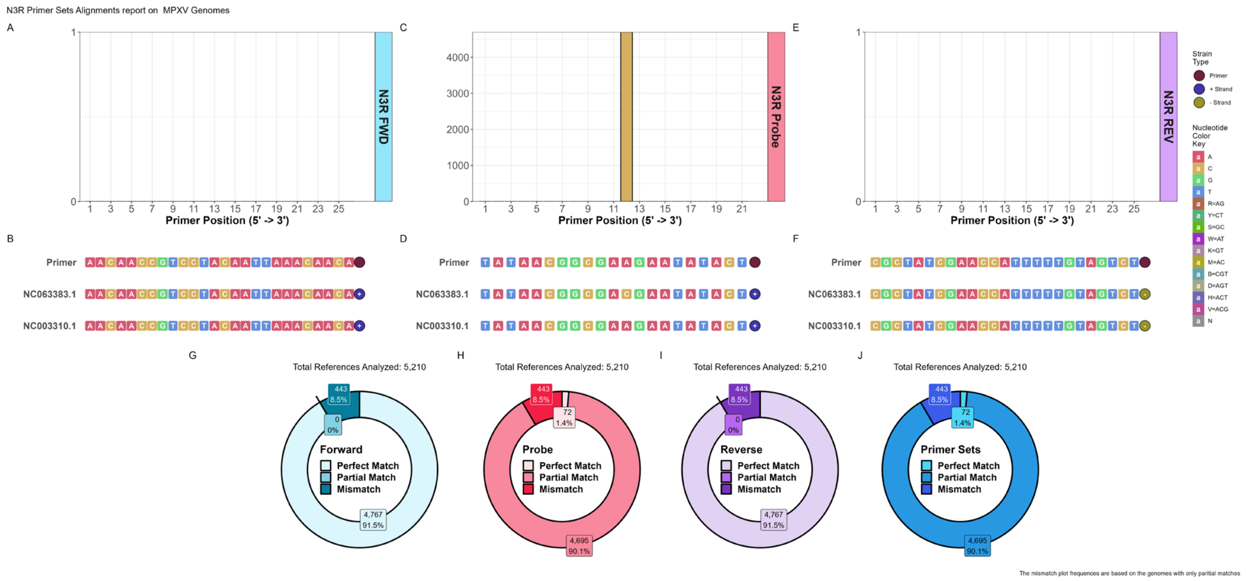

Supplementary Figure 16. The diagnostic plot of the forward, probe, and reverse primer alignment to 5,210 genomes of primer N3R. The partial alignment patterns across the forward, probe, and reverse sequences are highlighted in panels **A, C,** and **E,** respectively. The forward, reverse, and probe sequences that targeted the RefSeq MPXV Clade I (NC_003310.1) and Clade II (NC_063383.1) reference genome regions are illustrated in panels **B, D,** and **F,** respectively. Panels **G, H, I,** and **J** demonstrate the perfect, partial, and mismatch count and percentage for the forward, probe, reverse, and overall performance.

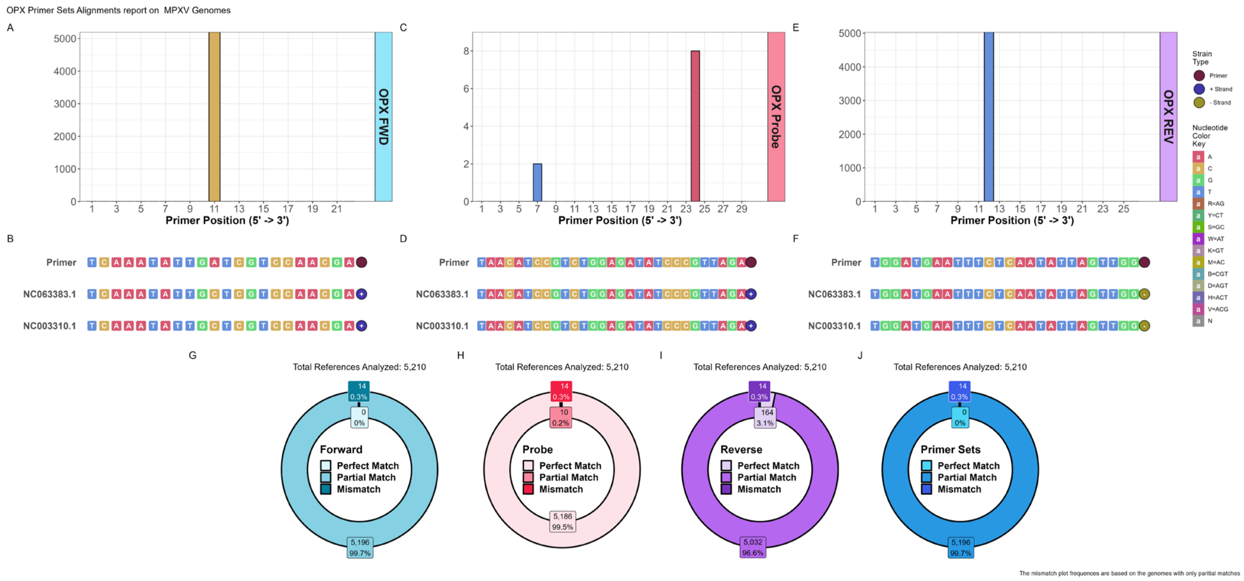

Supplementary Figure 17. The diagnostic plot of the forward, probe, and reverse primer alignment to 5,210 genomes of primer OPX. The partial alignment patterns across the forward, probe, and reverse sequences are highlighted in panels **A, C,** and **E,** respectively. The forward, reverse, and probe sequences that targeted the RefSeq MPXV Clade I (NC_003310.1) and Clade II (NC_063383.1) reference genome regions are illustrated in panels **B, D,** and **F,** respectively. Panels **G, H, I,** and **J** demonstrate the perfect, partial, and mismatch count and percentage for the forward, probe, reverse, and overall performance.

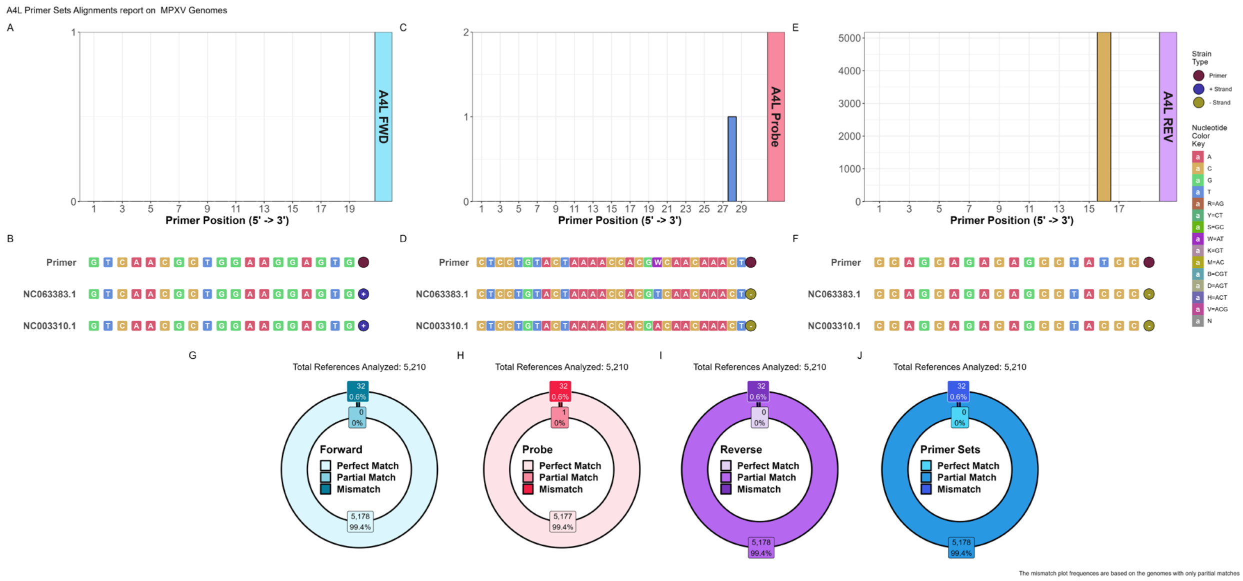

Supplementary Figure 18. The diagnostic plot of the forward, probe, and reverse primer alignment to 5,210 genomes of primer A4L. The partial alignment patterns across the forward, probe, and reverse sequences are highlighted in panels **A, C,** and **E,** respectively. The forward, reverse, and probe sequences that targeted the RefSeq MPXV Clade I (NC_003310.1) and Clade II (NC_063383.1) reference genome regions are illustrated in panels **B, D,** and **F,** respectively. Panels **G, H, I,** and **J** demonstrate the perfect, partial, and mismatch count and percentage for the forward, probe, reverse, and overall performance.

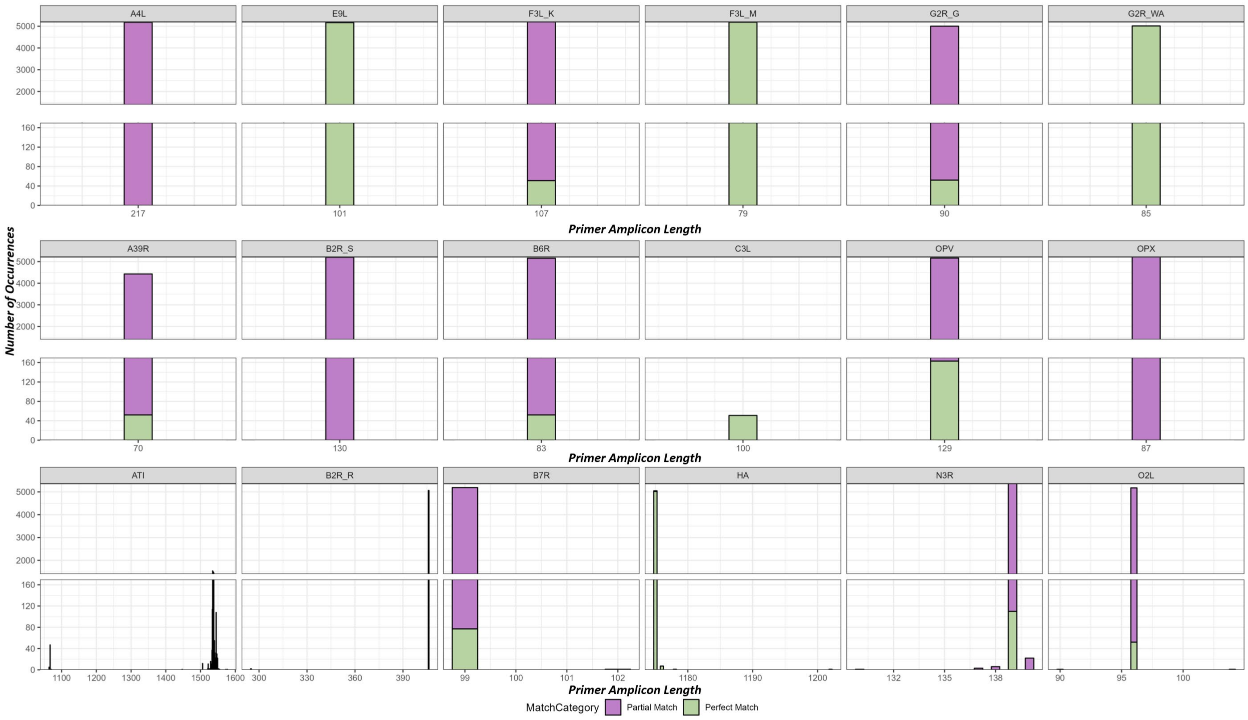

Supplementary Figure 19. Amplicon size distributions across 18 primer sets. Each subpanel is divided on the number of occurrences (y-axis) to provide zoomed view on 0-160, and 1,400-5,210 regions. The perfect and partial matches are colored green and purple, respectively.

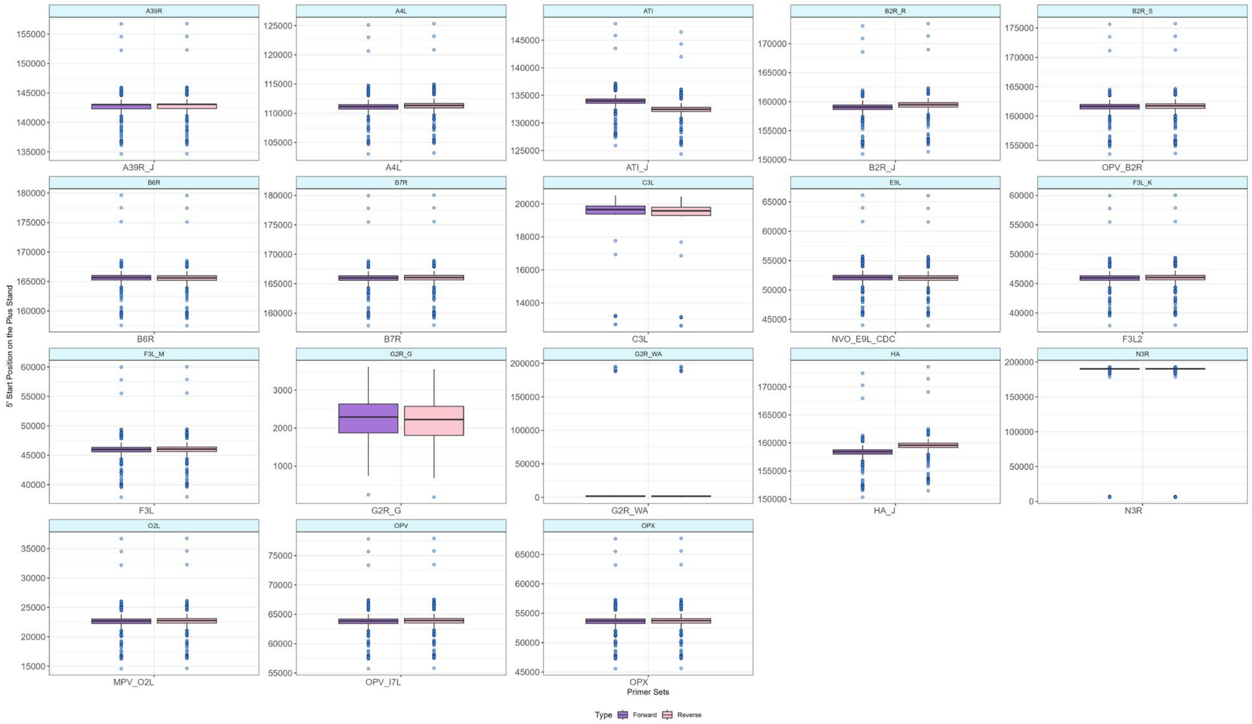

Supplementary Figure 20. The distribution of the start position of the forward and reverse primers of the 18 PCR primers. The forward and reverse primers are colored purple and pink, respectively.

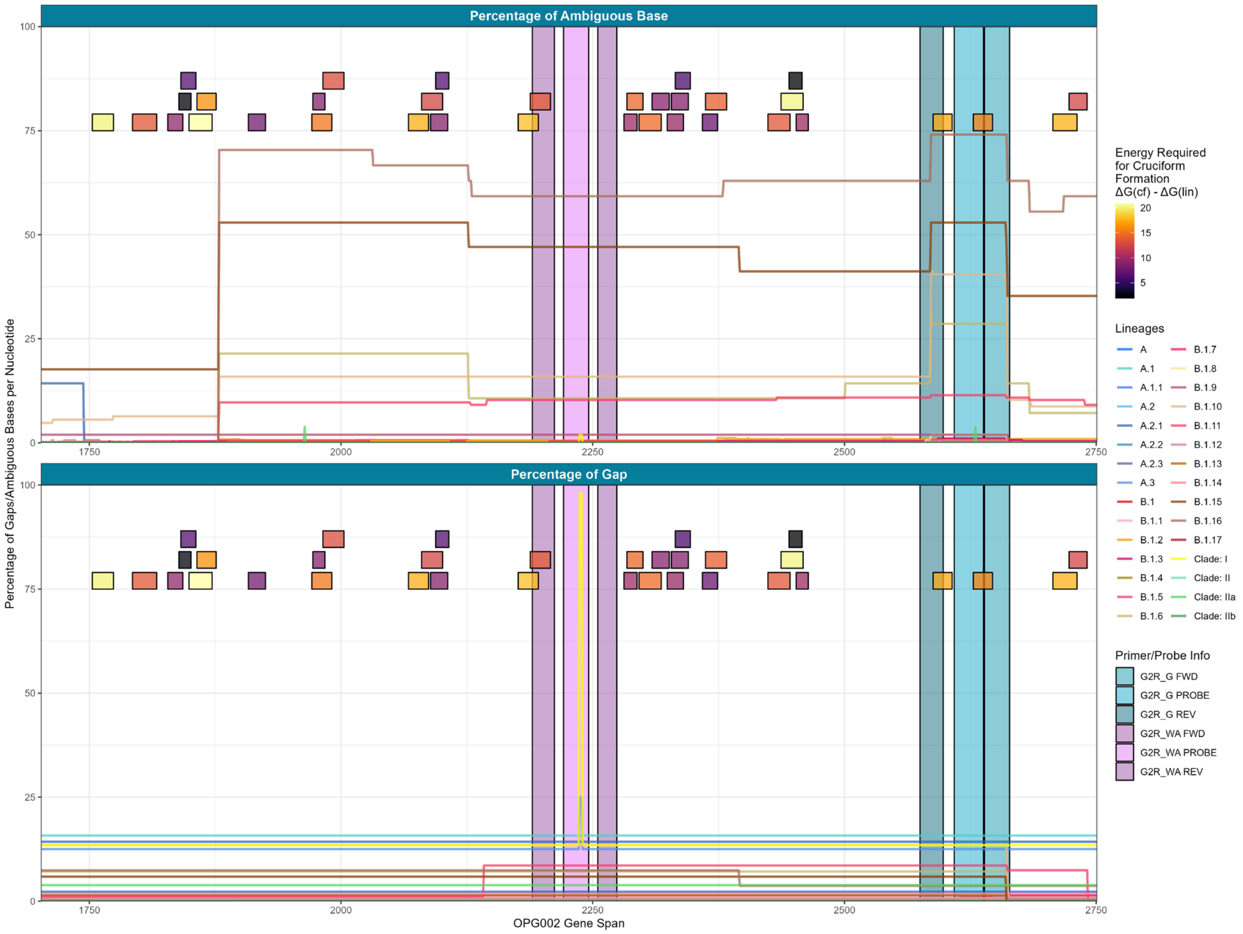

Supplementary Figure 21. Presence of ambiguous base ("N") and gap across the OPG002 that harbors G2R_WA and G2R_G. The vertical highlights show the range of the primers and probe for G2R_WA (purple) and G2R_G (blue). Each color blocks represents a detected inverted repeats identified with 0 or 1 mismatches. The yellower the color blocks, the higher the energy required for cruciform formation.

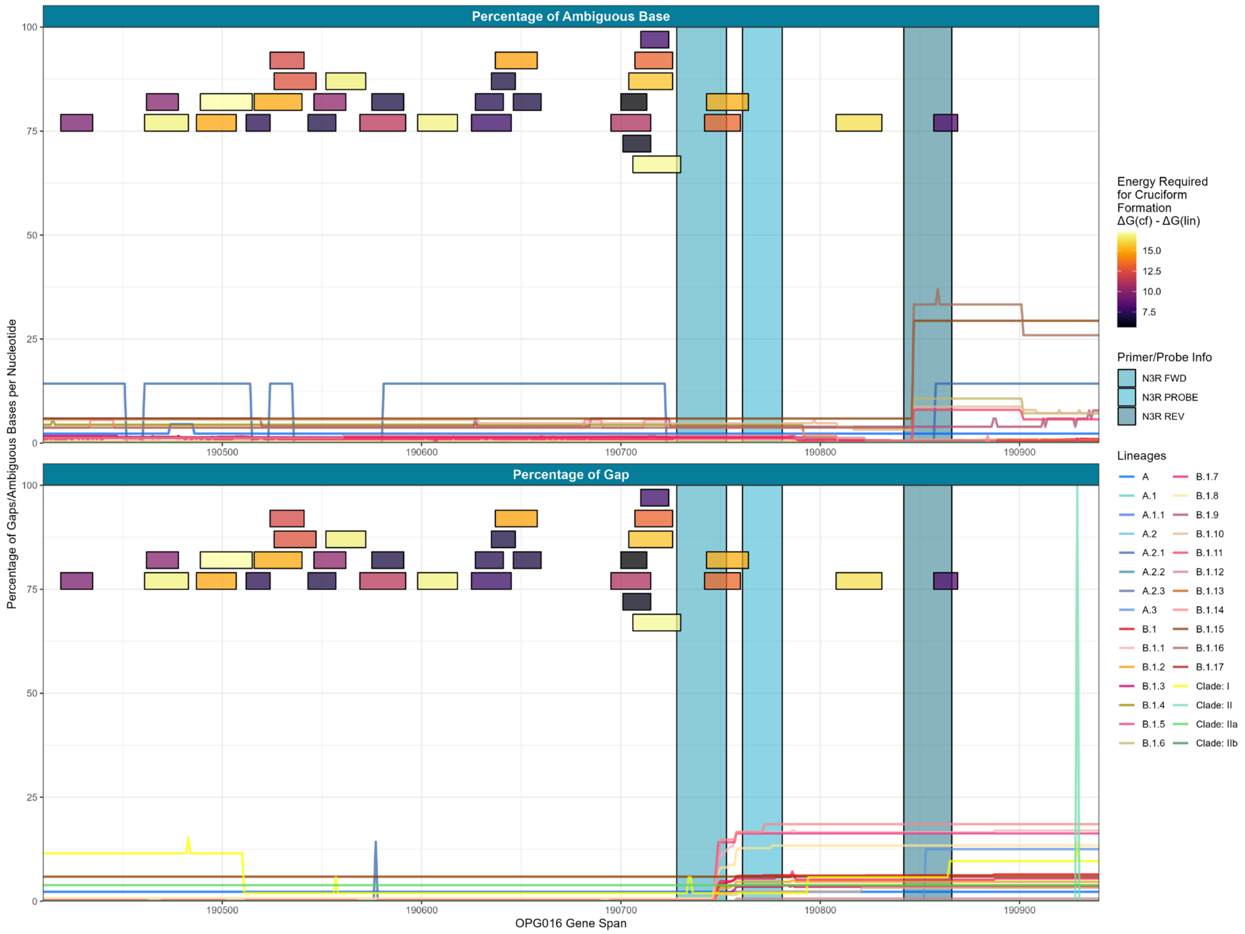

Supplementary Figure 22. Presence of ambiguous base ("N") and gap across the OPG016 that harbors N3R. The vertical highlights show the range of the primers and probe for N3R (blue). Each color blocks represents a detected inverted repeats identified with 0 or 1 mismatches. The yellower the color blocks, the higher the energy required for cruciform formation.

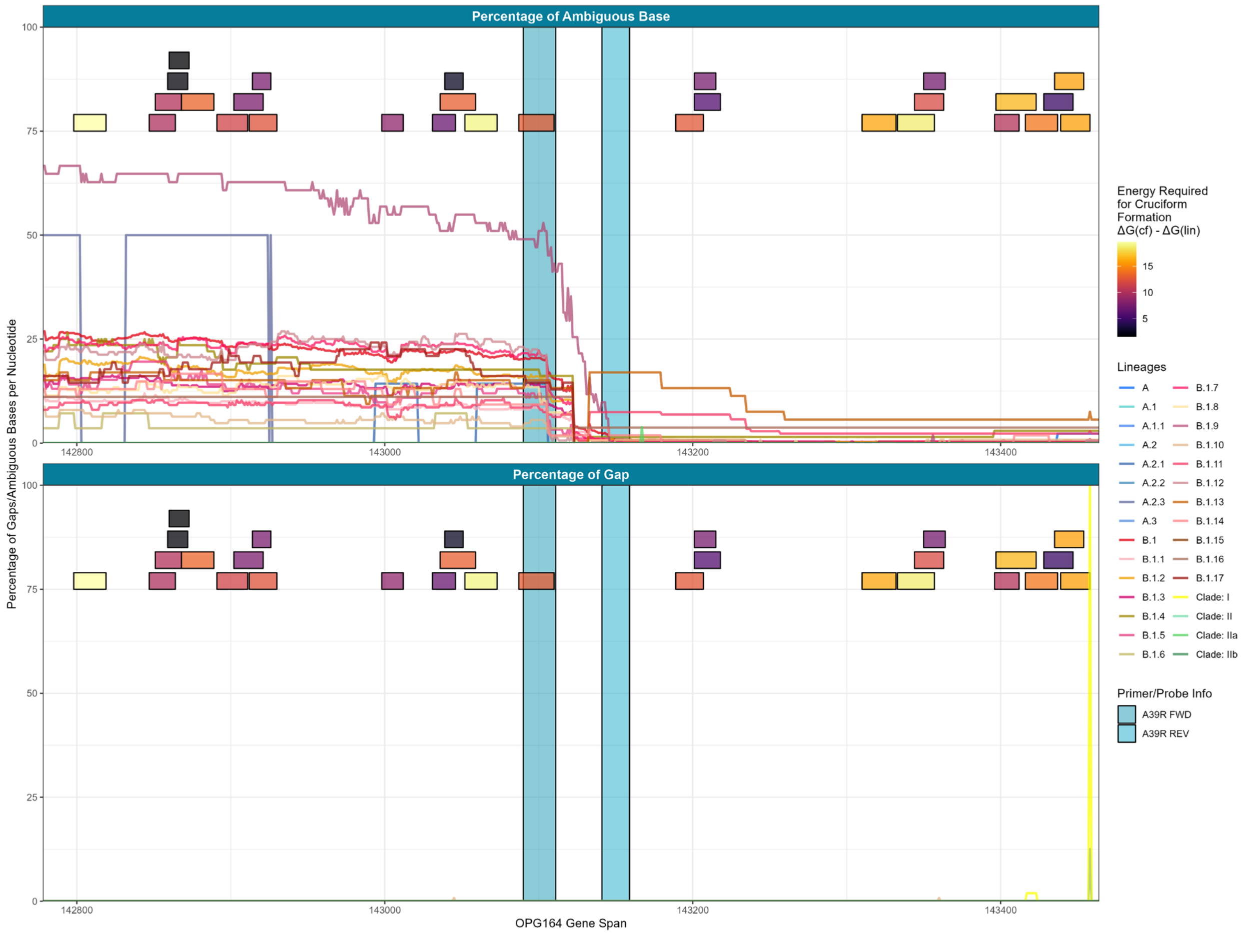

Supplementary Figure 23. Presence of ambiguous base ("N") and gap across the OPG164 that harbors A39R. The vertical highlights show the range of the primers and probe for A39R (blue). Each color blocks represents a detected inverted repeats identified with 0 or 1 mismatches. The yellower the color blocks, the higher the energy required for cruciform formation.
